# LDAPrototype: a model selection algorithm to improve reliability of latent Dirichlet allocation

**DOI:** 10.7717/peerj-cs.2279

**Published:** 2024-09-20

**Authors:** Jonas Rieger, Carsten Jentsch, Jörg Rahnenführer

**Affiliations:** Department of Statistics, TU Dortmund University, Dortmund, Germany

**Keywords:** Topic model, Stability, Similarity, Medoid, Clustering, Replications

## Abstract

Latent Dirichlet allocation (LDA) is a popular method for analyzing large text corpora, but it suffers from instability due to its reliance on random initialization. This results in different outcomes for replicated runs, hindering reproducibility. To address this, we introduce LDAPrototype, a new approach for selecting the most representative LDA run from multiple replications on the same dataset. LDAPrototype enhances the reliability of LDA conclusions by ensuring greater similarity between replications compared to traditional LDA runs or models chosen based on perplexity or NPMI. A key feature of LDAPrototype is its use of a novel model similarity measure called S-CLOP (Similarity of multiple sets by Clustering with LOcal Pruning). It is based on topic similarities, for which we compare the usage of measures like the thresholded Jaccard coefficient, cosine similarity, Jensen-Shannon divergence, and rank-biased overlap. The effectiveness of LDAPrototype is demonstrated through its application to six real datasets, including newspaper articles and tweets. The results show improved reproducibility and reliability in topic modeling outcomes. LDAPrototype’s approach is noteworthy for its practical applicability, comprehensibility, ease of implementation, and computational efficiency. Furthermore, the algorithm’s concept can be generalized to other topic modeling procedures that characterize topics through word distributions, making it a versatile tool in text data analysis.

## Introduction

Understanding unstructured data is generally a big challenge due to their complicated and typically non-standard forms. In particular, when dealing with text data, this is also the case due to the increasingly large volumes collected steadily these days. Text data is clearly one of the most frequent data types in the world today, and text mining tools have become very popular to analyze such data with the goal to address research questions from various fields of research.

Text data are often analyzed using so-called probabilistic topic models (Blei, 2012) and the latent Dirichlet allocation (Blei, Ng & Jordan, 2003) in particular. In the context of probabilistic topic models, the latent Dirichlet allocation (LDA) can be described as a successor to the probabilistic latent semantic indexing (Hofmann, 1999) and a predecessor to the correlated topic models (Blei & Lafferty, 2007). The LDA model has been extended by numerous features in the past years. Many of the implementations aim at the integration of meta variables such as author names in the author-topic model (Rosen-Zvi et al., 2004). Other models have attempted to integrate the temporal component of the modeled texts, *e.g.*, the continuous time dynamic topic models (Wang, Blei & Heckerman, 2008). In addition to the further development of the LDA, the structural topic model (Roberts et al., 2013) has proven to be another reliable model for the analysis of text corpora. Nevertheless, LDA still enjoys the highest popularity due to its simple implementation, flexible assumptions, and competitive results compared to other more refined, but also more complex methods.

In this paper, we propose the method LDAPrototype to improve the reliability of LDA results. In several use cases we show that single models are prone to misinterpretation because of potentially large differences in the results of LDA runs executed for the same text data. To do this, we define a similarity measure for LDA models, which we call S-CLOP (Similarity of multiple sets by Clustering with LOcal Pruning). This measure can be used to determine pairwise similarities of LDA models. To overcome the mentioned issue of reproducibility, which in turn leads to lower reliability, the method LDAPrototype selects the most representative run out of a set of LDA runs according to the novel S-CLOP criterion. This criterion is not based on likelihood-based measures as it is often the case. Instead, our goal is to determine the medoid of the models to increase the reliability of conclusions drawn from LDA results. In this context, we understand reliability as a measure to quantify the reproducibility of the results. That is, a highly reliable method should produce results that are as reproducible as possible, *i.e.,* replications of the model using the same parameters should result in as similar models as possible. We refer to the medoid as the model that agrees most on average with all other models. We call the resulting model the prototype, which is most similar to all other runs taken from the same modeling procedure obtained from he same text data.

A few approaches exist to make LDA results more reliable, but all of them have weaknesses, which are discussed in detail in ‘Methods and modifications of LDA to address the random initialization instability’. Often the modeling procedure itself is influenced in a way such that LDA loses its flexibility. Other methods do not search in the whole space of possible models, which may lead to non-optimal results. To address these weaknesses, our approach does explicitly not affect the modeling procedure itself and is exclusively based on replicated runs of the same topic model.

We do not use likelihood-based measures, because there is already good comparative work in this field, *e.g.*, by Griffiths & Steyvers (2004), and Chang et al. (2009), who showed that these measures do not correlate well with the human perception of consistent topics. Nevertheless, we evaluate the ability of our method to find reliable models in comparison to the relatively popular measure of perplexity and the well-known coherence measure NPMI. However, the quality of a topic model, which is mainly argued from a high interpretability of the results, is not within the scope of this paper. Subsequent studies with human judgments are needed for this. Instead, the main goal is to address the issue of reliability caused by the instability inherent to the LDA through a model selection criterion to increase the reliability of the results. For this purpose, we define reliability as a measure of the degree of reproducibility. We call a model highly reliable if it produces very similar results under the same model parameter settings, such that the resulting models are only differing due to the random initializations. In ‘Methods’, we formally define a measure of reliability, for which we then show that our new method LDAPrototype achieves higher scores than common selection methods based on perplexity or NMPI.

High reliability can be considered as the basis and crucial prerequisite to also obtain good results in terms of quality. Thus, we do not follow the classical information retrieval approach, but focus first on increasing reliability of results in order to finally obtain topics that can be interpreted particularly well, which then would correspond to model’s quality. We intentionally do not speak of an improvement in stability here, because the LDA method, which is intrinsically depending on a random initialization, is not changed, and thus is just as (in)stable as before. Our proposed selection algorithm based on replicated runs on the same text data to obtain the most representative LDA run, on the contrary, provides an improvement of the reliability, which is measured by the degree of reproducibility quantified by our reliability score.

## Related Work

Text data are usually organized in large corpora, where each corpus consists of a collection of texts, often also denoted as documents or articles. Each text can be considered as a sequence of tokens of words of the same length as the given text. In common notation token means an individual word at a specific place in the text and the set of words is used synonymously with vocabulary. We refer to these terms in the following to introduce the methodology of LDA as basis for our novel method LDAPrototype. Portions of this text were previously published as part of a preprint (Rieger, 2022).

### Topic models

Propably the most well-known topic model is the classical LDA (Blei, Ng & Jordan, 2003), which we explain in ‘Latent Dirichlet allocation’ in detail. It can be seen as a refinement of probabilistic latent semantic analysis/indexing (Hofmann, 1999), which itself is a refinement of the latent semantic indexing (Deerwester et al., 1990). Consequently, the structural topic model (Roberts et al., 2013) can again be seen as a generalization of LDA. Concretely, it allows correlations between topics as well as the possibility to include metadata in the modeling of topic and word distributions.

Along with the popularity of neural networks, neural topic models or topic models based on word embeddings (Mikolov et al., 2013, cf.) such as the embedded topic model (Dieng, Ruiz & Blei, 2020) emerged. However, due to the rapid innovation cycles, these models have found little popularity and application. Moreover, their architecture prevents them from being directly comparable with traditional probabilistic topic models, meaning that evaluation measures developed for probabilistic topic models are not well suited to determine quality of such models (Hoyle et al., 2022).

In recent years, following the development of the transformer architecture (Vaswani et al., 2017) (not only) for topic models, the focus shifted to enhancements of models based on language models. Examples of such models are GRETEL (Xie et al., 2022), STELLAR (Eklund & Forsman, 2022) and the quite popular BERTopic (Grootendorst, 2022), the latter of which can be used extremely flexibly due to its modular combination of language models, clustering techniques, and scoring functions.

However, these novel transformer-based language models, which can be considered as superposed neural topic models (Lim & Lauw, 2023), allow more than just the possibility of being used directly for modeling. Rather, language models offer possibilities (cf. Sia, Dalmia & Mielke, 2020; Mu et al., 2024) to use them as a complementary evaluation option (Stammbach et al., 2023).

In fact, a major problem in topic model research is the lack of a satisfying task-independent evaluation of topic models. In this regard, instead of using (just) likelihood-based measures (Chang et al., 2009), topic models should always be compared using task-based measures (cf. Grimmer & Stewart, 2013; Doogan & Buntine, 2021). In addition, an automated evaluation often does not provide trustworthy results. Hoyle et al. (2021) show that coherence-based measures sometimes provide incoherent results and they argue that automated measures require ongoing human re-assessment in order to remain reliable. Overall, this shows that there is not a one-fits-it-all solution for all areas where topic models are used (cf. Ethayarajh & Jurafsky, 2020).

In practice, it can be seen that LDA is still very popular and frequently used in state-of-the-art studies (cf. Batool & Byun, 2024; Muthusami et al., 2024), so that further developments of this methodology, which do not change the model assumptions and modeling itself, continue to be highly relevant.

### Latent Dirichlet allocation

The method we propose is based on the LDA (Blei, Ng & Jordan, 2003) estimated by a collapsed Gibbs sampler (Griffiths & Steyvers, 2004). The LDA assumes distributions of latent topics for each text. If *K* denotes the total number of modeled topics, the set of topics is given by ***T*** = {*T*_1_, …, *T*_*K*_}. We define ${W}_{n}^{(m)}$ as a single token at position *n* in text *m*. The set of possible tokens is given by the vocabulary ***W*** = {*W*_1_, …, *W*_*V*_} with *V* = |***W***|, the vocabulary size. Then, let

\begin{eqnarray*}{\mathbi{D}}^{(m)}= \left( {W}_{1}^{(m)},\ldots ,{W}_{{N}^{(m)}}^{(m)} \right) ,~m=1,\ldots ,M,\quad {W}_{n}^{(m)}\in \mathbi{W},~n=1,\ldots ,{N}^{(m)}& \end{eqnarray*}

be text (or document) *m* of a corpus consisting of *M* texts, each text of length *N*^(*m*)^. Topics are referred to as ${T}_{n}^{(m)}$ for the topic assignment of token ${W}_{n}^{(m)}$. Then, analogously the topic assignments of every text *m* are given by

\begin{eqnarray*}{\mathbi{T}}^{(m)}= \left( {T}_{1}^{(m)},\ldots ,{T}_{{N}^{(m)}}^{(m)} \right) ,~m=1,\ldots ,M,\quad {T}_{n}^{(m)}\in \mathbi{T},~n=1,\ldots ,{N}^{(m)}.& \end{eqnarray*}

when ${n}_{k}^{(mv)},$k =1 , …, *K*, v =1 , …, *V* describes the number of assignments of word *v* in text *m* to topic *k*, we can define the cumulative count of word *v* in topic *k* over all documents by ${n}_{k}^{(\bullet v)}$ and, analogously, the cumulative count of topic *k* over all words in document *m* by ${n}_{k}^{(m\bullet )}$, while ${n}_{k}^{(\bullet \bullet )}$ indicates the total count of assignments to topic *k*. Then, let

\begin{eqnarray*}{\mathbi{w}}_{k}={ \left( {n}_{k}^{(\bullet 1)},\ldots ,{n}_{k}^{(\bullet V)} \right) }^{T}\in {\mathbb{N}}_{0}^{V},~k=1,\ldots ,K& \end{eqnarray*}

denote the vector of word counts for topic *k*.

Using these definitions, the underlying probability model (Griffiths & Steyvers, 2004) can be written as

\begin{eqnarray*}{W}_{n}^{(m)}\mid {T}_{n}^{(m)},{\phi }_{k}\sim ~~\text{Discrete}({\phi }_{k}),\quad {\phi }_{k}\sim ~~\text{Dirichlet}(\eta ),& \nonumber\\\displaystyle {T}_{n}^{(m)}\mid {\theta }_{m}\sim ~~\text{Discrete}({\theta }_{m}),\quad {\theta }_{m}\sim ~~\text{Dirichlet}(\alpha ).& \end{eqnarray*}

For a given parameter set {*K*, *α*, *η*}, LDA assigns one of the *K* topics to each token. Here *K* denotes the number of topics and *α*, *η* are parameters of a Dirichlet distribution defining the type of mixture of topics in every text and the type of mixture of words in every topic. Higher values for *α* lead to a more heterogeneous mixture of topics whereas lower values are more likely to produce less but more dominant topics per text. Analogously, *η* controls the mixture of words in topics. Although the LDA permits *α* and *η* to be vector valued (Blei, Ng & Jordan, 2003), they are usually chosen symmetric because typically the user has no a-priori information about the topic distributions *θ* and word distributions *ϕ*.

Topic distributions per text *θ*_*m*_ = (*θ*_*m*,1_, …, *θ*_*m*,*K*_)^*T*^ ∈ (0, 1)^*K*^ and word distributions per topic *ϕ*_*k*_ = (*ϕ*_*k*,1_, …, *ϕ*_*k*,*V*_)^*T*^ ∈ (0, 1)^*V*^ can be estimated through the collapsed Gibbs sampler procedure (Griffiths & Steyvers, 2004) by

\begin{eqnarray*}{\hat {\theta }}_{m,k}= \frac{{n}_{k}^{(m\bullet )}+\alpha }{{N}^{(m)}+K\alpha } \quad \text{and}\quad {\hat {\phi }}_{k,v}= \frac{{n}_{k}^{(\bullet v)}+\eta }{{n}_{k}^{(\bullet \bullet )}+V\eta } .& \end{eqnarray*}

### Methods and modifications of LDA to address the random initialization instability

Inferring LDA using Gibbs sampling is sensitive to the initial assignments, that are often chosen as random, and the reassignment is based on the conditional distributions, which leads to different results in multiple LDA runs for fixed parameters. This instability of LDA leads to a lack of reliability of the modeling results. This fact is rarely discussed in applications (Agrawal, Fu & Menzies, 2018), although several approaches have been proposed to encounter this problem as discussed in the following.

#### Parameter tuning

Agrawal, Fu & Menzies (2018) propose a new algorithm LDADE (LDA Differential Evolution) which automatically tunes the parameters of LDA in order to optimize topic similarity in replications using a differential evolution algorithm. This results in a set of input parameters *K*, *α* and *η* which perform best on the given data with respect to reliability. This procedure does not increase the reliability for a given parameter set, but tries to find the parameter set that produces the most reliable results. So this method aims for parameter optimization. However, it is likely that the tuning algorithm is biased to select parameters that result in systematically better reliability values independent of the underlying dataset, *e.g.*, for low *α* and *η* parameters. In many applications, it is of interest to choose the parameters of the LDA reasonably based on external knowledge. Accordingly, our method focuses on the optimization of the reliability for fixed parameter sets instead of parameter optimization as performed by LDADE.

#### Selection and averaging algorithms

Another option is to apply a selection criterion to a set of models (Wallach et al., 2009). The selection can be done by optimizing perplexity (Blei, Ng & Jordan, 2003), a performance measure for probabilistic models to estimate how well new data fit into the model (Rosen-Zvi et al., 2004). Alternatively, Nguyen, Boyd-Graber & Resnik (2014) proposed to average stages of the Gibbs sampling procedure. They present different variations to average iteration steps and show that their approach leads to an increase of perplexity. Averaging LDA models comes with the drawback that one only receives averaged topic proportions, but no specific topic assignment per token. In addition, it was shown that likelihood-based measures like perplexity are negatively correlated with human judgments on topic quality (Chang et al., 2009). Instead, optimizing the semantic coherence of topics should be the aim for a selection criterion. Chang et al. (2009) provide a validation technique called Word/Topic Intrusion (implemented in Koppers et al., 2020) which depends on a human coding process. Automated measures to select the best LDA regarding coherence can be transferred from the topic coherence measure (Mimno et al., 2011; Stevens et al., 2012), but there is no stable and validated aggregation technique of this type of topic quality measure for the results of LDA runs. Instead, Röder, Both & Hinneburg (2015) introduce some other measures for quantifying topic quality based on coherence measures, such as the normalized pointwise mutual information (NPMI), for which Xing, Paul & Carenini (2019) show, that it outperforms other automatically calculated quality measures regarding correlation to human scores for topic quality. For this reason in ‘Increase of reliability’, we compare the still most popular selection measure perplexity and the coherence measure NPMI in terms of improving the reliability of the selected LDA models.

#### Manual approaches and reasonable initialization

Maier et al. (2018) aim for increasing both, reliability and interpretability of the final model simultaneously. Therefore, they maximize topic similarity as well as topic coherence, but discover that standard metrics in general do not perform well in increasing interpretability. Instead, manual approaches as the mentioned intruder validation technique proposed by Chang et al. (2009) are essential. Maier et al. (2018) propose to increase reliability of LDA by initializing topic assignments of the tokens reasonably, *e.g.*, using co-occurrences of words (Newman, Bonilla & Buntine, 2011). This initialization technique has the drawback that the model is restricted to a subset of possible results.

#### Model modifications

There is also a modification of the implementation of LDA that aims to reduce instability. GLDA (Granulated LDA) was proposed by Koltcov et al. (2016) and is based on a modified Gibbs Sampler. The idea of the algorithm is that tokens that are closer to each other are more likely to be assigned to the same topic. The authors show that their algorithm performs comparably well with standard LDA regarding interpretability. Moreover, it leads to more stable results. However, their study is based on only three LDA runs and the implementation is not publicly available. Thus, a validation of this method on other datasets or with larger numbers of replications is pending.

## Contributions

In this work, we propose the novel selection algorithm LDAPrototype based on the tailored similarity measure S-CLOP for LDA models. Thus, our contribution is two-fold. The S-CLOP measure is able to assess the stability of LDA with clustering techniques applied to replicated LDA runs. High stability corresponds to high reliability of findings based on stable models in the sense of improving reproducibility. We introduce a new automated method of clustering topics, more precisely a pruning algorithm for results of hierarchical clustering, based on the optimality criterion that for clustered results of replicated LDA runs, in the ideal case each cluster should contain exactly one topic of every replication of the modeling procedure. This results in our novel tailored similarity measure S-CLOP (Similarity of multiple sets by Clustering with LOcal Pruning) for LDA runs. We demonstrate the potential of this measure to improve reliability by applying it to example corpora. Based on the newly proposed similarity measure S-CLOP, we propose a combination of a repetition strategy and selection criterion to increase the reliability of findings from LDA models leading to the LDAPrototype algorithm. We show, that it outperforms perplexity and NPMI regarding a reliability measure that is based on LDA similarities.

## Methods

We introduce the new method LDAPrototype that selects the medoid of a number of LDA runs. The selection is achieved by choosing the model that maximizes the mean pairwise S-CLOP value to all other LDA runs. For assessing similarities of LDA models using our novel S-CLOP measure, also an adequate similarity measure for topics is required. We define a more robust version of the Jaccard coefficient in the sense that not all words are considered as relevant for each topic. In ‘Results’, the selection algorithm LDAPrototype is applied to six example corpora to assess the increase in reliability of findings from LDA models. In ‘Comparison of the implemented similarity measures’, we also present other implemented similarity measures, which are compared to our thresholded Jaccard coefficient regarding reliability gain and computation time.

The introduced methods have been implemented as R package (Rieger, 2020) on CRAN and are available at https://github.com/JonasRieger/ldaPrototype as continuously developing GitHub repository.

### LDAPrototype: a new selection algorithm for LDA models

We propose the novel selection algorithm LDAPrototype to improve the reliability of LDA results. For this, we define the reliability score *rs* for a set of *L* LDAs based on their mean similarities $\bar {{s}_{1}},\ldots ,\bar {{s}_{L}}$ as

(1) \begin{eqnarray*}rs\,(\bar {{s}_{1}},\ldots ,\bar {{s}_{L}}):= \frac{1}{L} \sum _{l=1}^{L}\bar {{s}_{l}}, \bar {{s}_{l}}\in [0,1],\end{eqnarray*}

where the mean similarities $\bar {{s}_{l}},$l =1 , …, *L* have to be determined by a model similarity measure yet to be defined. Our selection algorithm aims for maximizing this reliability score *rs*, which measures how reproducible LDA results are in a given setting with fixed parameters. Our algorithm is motivated by an increase in reliability (cf. ‘Introduction’) and selects from a set of LDA models the model that is most similar on average to all other runs. The approach is similar to the choice of the median in the one-dimensional space. In the multidimensional space, this choice is called medoid and differs from a centroid in the sense that it is not obtained by model averaging, but by model selection. There are methods of model averaging for LDA (cf. ‘Methods and modifications of LDA to address the random initialization instability’), but these have the disadvantage that properties of a single run, such as the assignments of individual tokens to topics, are lost. The proposed selection algorithm preserves this information because it does not influence the modeling itself. The LDAPrototype procedure selects one single model from the set of candidate models.

**Figure 1:**
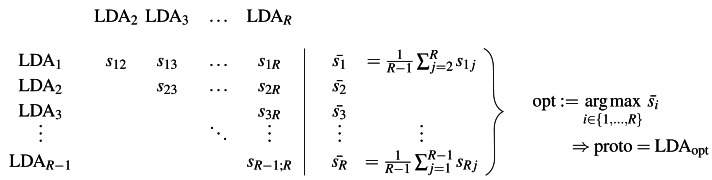
Schematic representation of the determination of a prototype based on a set of LDA models.

 
Figure 1 shows schematically the determination of the prototype, and the corresponding procedure is presented in Algorithm 1 . The main idea of the method is to calculate all pairwise similarities of the *R* LDAs and determine the model that maximizes this similarity. Besides the candidate set of LDAs {LDA_1_, …, LDA_*R*_}, a similarity measure for LDA models is needed to obtain the symmetric model similarity scores *s*_*ij*_ = *s*_*ji*_ in Fig. 1 and Algorithm 1 . For this, in ‘S-CLOP: a new similarity measure for LDA models’, we propose a novel tailored model similarity measure called S-CLOP, which in turn requires the choice of a topic similarity measure. In the following section ‘Thresholded version of the Jaccard coefficient: a similarity measure for topics’, we propose a default measure for computing these topic similarities. Through these definitions, we are then able to determine the prototype with respect to Fig. 1. In Appendix A, we discuss other popular measures for computing topic similarities, which we also compare to our proposed Jaccard coefficient from Eq. (3) in ‘Comparison of the implemented similarity measures’.

_______________________ 
Algorithm 1 Selecting the medoid of a number of LDA runs______________________ 
Require: A set of R LDA models 
Ensure: The LDA with maximal mean pairwise similarity to all other LDA 
     runs 
  1:  for i = 1 to R − 1 do 
 2:       for j = i + 1 to R do 
 3:            Calculate pairwise similarity of LDAs ldai and ldaj using a similarity 
     measure sim: sij = sim(ldai,ldaj) 
  4:       end for 
 5:  end for 
 6:  Determine ˉ si =   1 __ 
R−1 ∑ 
   j⁄=i sij, i = 1,...,R 
 7:  Determine proto = ldaopt with opt = arg max 
 i∈{1,...,R} ˉ si 
  8:  return proto_________________________________________________________________________________    

### Thresholded version of the Jaccard coefficient: a similarity measure for topics

A similarity between two topics can be calculated based on the corresponding vectors of counts or set of words. We build on the well established Jaccard coefficient (Jaccard, 1912) and introduce a more robust thresholded version. Its general form is given by

(2) \begin{eqnarray*}\text{Jaccard}(A,B)= \frac{{|}A\cap B{|}}{{|}A\cup B{|}} ,\end{eqnarray*}

where *A*, *B* are sets of words.

Suppose we have a text corpus and we estimate a topic model with LDA, with parameters *α*, *η* and *K* topics. This is done *R* times independently leading to a set of *N* = *RK* topics in total. Then, referring to ‘Latent dirichlet allocation’,

\begin{eqnarray*}{\mathbi{w}}^{(r)}= \left( {\mathbi{w}}_{1}^{(r)},\ldots ,{\mathbi{w}}_{K}^{(r)} \right) \in {\mathbb{N}}_{0}^{V\times K},r=1,\ldots ,R \end{eqnarray*}

denotes the matrix of word counts per topic in the *r*-th replication. Then, for a given lower bound ***c*** = (*c*_1_, …, *c*_*N*_) and for two topics (*i*, *j*) represented by their word count vectors

\begin{eqnarray*}{\mathbi{w}}_{i},{\mathbi{w}}_{j}\in \left\{ {\mathbi{w}}_{1}^{(1)},\ldots ,{\mathbi{w}}_{K}^{(1)},{\mathbf{w}}_{1}^{(2)},\ldots ,{\mathbi{w}}_{K}^{(2)},\ldots ,{\mathbi{w}}_{1}^{(R)},\ldots ,{\mathbi{w}}_{K}^{(R)} \right\} \end{eqnarray*}

our thresholded version of the Jaccard coefficient is calculated by

(3) \begin{eqnarray*}\text{tJacc}({\mathbi{w}}_{i},{\mathbf{w}}_{j})= \frac{\sum _{v=1}^{V}{1}_{ \left\{ {n}_{i}^{(\bullet v)}> {c}_{i}\wedge {n}_{j}^{(\bullet v)}> {c}_{j} \right\} }}{\sum _{v=1}^{V}{1}_{ \left\{ {n}_{i}^{(\bullet v)}> {c}_{i}\vee {n}_{j}^{(\bullet v)}> {c}_{j} \right\} }} .\end{eqnarray*}

Reasonable choices for the threshold vector ***c*** = (*c*_1_, …, *c*_*N*_)^*T*^ ∈ ℕ^*N*^ are an equal absolute lower bound *c*_abs_ for all words, or a topic-specific relative lower bound *c*_rel_ (see also Table 1). A combination of both can be defined by

\begin{eqnarray*}{c}_{l}=\max \nolimits \{ {c}_{\text{abs}},{c}_{\text{rel}}{n}_{l}^{\bullet \bullet }\} , \end{eqnarray*}

where *l* = 1, …, *K*, …, 2*K*, …, *RK* = *N* and *c*_abs_ ∈ ℕ_0_, *c*_rel_ ∈ [0, 1]. To ensure that always enough words per topic are taken, the similarity measure is additionally implemented with a parameter that controls that at least a fixed user-defined number of the most frequent words assigned to the topic are considered.

**Table 1:** Toy example: Assignment counts of two topics and calculation of thresholded version of the Jaccard (tJacc) coefficient for *c*_rel_ = 0.002.

	***w***_1_	***w***_2_	∧	∨	
trump	1,668	2,860	1	1	$\begin{array}{@{}r@{}} \displaystyle \text{vocabulary size}~~V=11,\\ \displaystyle \text{relative limit}~~{c}_{\text{rel}}=1/500\\ \displaystyle \Rightarrow \mathbi{c}=({n}_{1}^{\bullet \bullet },{n}_{2}^{\bullet \bullet })^{T}/500\\ \displaystyle =(4459,6287)^{T}/500\\ \displaystyle =(8.92,12.57)^{T}.\\ \displaystyle \\ \displaystyle \text{tJacc}({\mathbi{w}}_{1},{\mathbi{w}}_{2})= \frac{5}{10} . \end{array}$
trumps	446	854	1	1	
president	91	876	1	1	
donald	259	693	1	1	
news	695	0	0	1	
said	500	0	0	1	
election	8	474	0	1	
will	0	462	0	1	
women	397	53	1	1	
debate	394	11	0	1	
sarcastic	1	4	0	0	
Σ	4,459	6,287	5	10	

The interpretation of this tJacc coefficient is the following. It is defined as the ratio of the numbers of the intersection and the union of the words of two topics, but a word is only considered if the number of its occurrences in the texts exceeds the topic-specific threshold. In other words, we first restrict ourselves to the most relevant words per topic with respect to the number of assignments, to get rid of heavy tailed word lists. Then the resulting subsets of words are used to measure similarity of topics using the standard Jaccard coefficient. To be clear: even for a choice of *c*_rel_ = 0, the similarities of the topics would not (necessarily) all equal 1. That is because for the calculation not the estimators ${\hat {\phi }}_{k,v}$, but the numbers of actual assignments ${n}_{i}^{(\bullet v)}$ are used.

We demonstrate how the measure is calculated with a small toy example.

In Table 1, for eleven selected words the counts of assignments over all articles for the two topics ***w***_1_ and ***w***_2_ are given. We use the relative lower bound with *c*_rel_ = 1/500. In the analysis presented in ‘Results’, we mainly also use *c*_rel_ = 1/500, which leads to around 100 important words per topic in the setting of the *usatoday* dataset. The last two columns indicate whether the corresponding word belongs to the thresholded intersection or union, respectively. For example, the word *election* does not belong to the intersection because its count is below the topic-specific (relative) threshold of at least nine assignments to the topic belonging to ***w***_1_. The ratio of the number of entries in the third and the fourth column results in the similarity $\text{tJacc}({\mathbi{w}}_{1},{\mathbi{w}}_{2})= \frac{5}{10} =0.5$ of the two given topics.

### S-CLOP: a new similarity measure for LDA models

We introduce the new similarity measure S-CLOP for comparing different LDA runs consisting of topics that are represented by word count vectors. The pairwise distances are calculated with the tJacc coefficient in Eq. (3). However, the measure can be applied using any appropriate distance respectively similarity measure, see Appendix A. We use S-CLOP as pairwise model similarity measure in the LDAPrototype procedure, see also Fig. 1 and Algorithm 1 .

The general idea of the measure S-CLOP is the following. First, join all considered LDA runs to one overall set, then cluster the topics with subsequent local pruning, and then check how many members of the original different LDA runs are contained in the resulting clusters. Then the deviation from the perfect situation of one representative from each LDA run, i.e. that every topic is present in all LDA runs, is quantified. In our selection algorithm always two LDA models are compared. Two models are very similar, if always one topic of the first model is clustered together with one topic from the other model. High S-CLOP similarity values suggest that many topics can be identified that have a representative in each LDA run.

For the initial clustering step, we use hierarchical clustering with complete linkage (Hastie, Tibshirani & Friedman, 2009 pp. 520–525). According to Zhao, Karypis & Fayyad (2005) average linkage or Ward’s method obtain superior results for the case of text clustering. We also tried single linkage, average linkage and Ward’s method in the present case, and in the package the user is also offered, in principle, the possibility of calculation using these link methods. In fact, for the selection algorithm presented here, there is little difference in which of the linkage methods is used due to the characteristic of combining pairwise comparison with local-optimal pruning. We prefer complete linkage over single or average linkage because it uses the maximum distance between topics to identify clusters. This is consistent with our aim of identifying highly homogeneous groups. Of course, topic similarities must first be transformed to distances to apply hierarchical clustering.

#### Measuring disparity of LDA runs

Consider a cluster (respectively a group) *g* of topics, after clustering *R* LDA runs in one joint cluster analysis, using all *R*⋅*K* topics from all runs. The goal is to quantify the deviation from the desired situation that each run is represented exactly once in *g*, which can be measured using disparity. The vector ${\mathbi{t}}^{(g)}=({t}_{1}^{(g)},\ldots ,{t}_{R}^{(g)})^{T}\in {\mathbb{N}}_{0}^{R}$ contains the number of topics that belong to the different LDA runs. Then, we define the disparity measure

(4) \begin{eqnarray*}U(g):= \frac{1}{R} \sum _{r=1}^{R}{|}{t}_{r}^{(g)}-1{|}\cdot \sum _{r=1}^{R}{t}_{r}^{(g)}.\end{eqnarray*}

The first factor ${|}{t}_{r}^{(g)}-1{|}$ measures the deviation from the best case of exactly one topic per run in *g*. The second factor determines the number of members in the cluster and is required to penalize large clusters. Without this adjustment, the algorithm presented below for minimizing the sum of disparities would prefer one large cluster over a number of small clusters. In particular, without the second term, joining two perfect clusters as well as splitting one perfect cluster in two clusters would result in the same value for the mean disparity (*R*/*R* = 1), and we prefer the second situation, where two different topics from one run are not clustered together. The disparity of one overall cluster *g* containing all topics, *e.g*. defined by the root of a dendrogram, is given by *U*(*g*) = (*K* − 1)⋅*N*.

#### Finding the best cluster result by minimizing average disparity

The goal is to minimize the sum of disparities *U*(*g*) over all groups *g* ∈ *G* of a cluster result. Hierarchical clustering of all *N* objects (topics) provides cluster results with 1, …, *N* clusters. A common approach is to globally cut the dendrogram according to a target value. Here, we propose to prune the resulting dendrogram locally to obtain the final clusters. The pruning algorithm requires as input a hierarchical clustering result and minimizes the sum of disparities, with respect to the dendrogram structure, *i.e.,*

(5) \begin{eqnarray*}{U}_{\Sigma }(G):=\sum _{g\in G}U(g)\rightarrow \min \nolimits ,\end{eqnarray*}

where *G* is a set of clusters (of topics), and the set of all topics is a disjoint union of the members of the single clusters *g* ∈ *G*.

Denote by *G*^∗^ the optimal set of clusters resulting from splits identified from the dendrogram, and by *U*^∗^: = *U*_Σ_(*G*^∗^) the corresponding minimal sum of disparities. The root of the dendrogram contains (as a disjoint union) the members of the two nodes obtained by the first split. Likewise, iteratively, each node contains (again, as a disjoint union) the members of the two nodes on a clustering level one step below this specific node, as denoted in Algorithm 2 by node.left and node.right. The optimal sum *U*^∗^ can be calculated recursively with Algorithm 2 .

 
_______________________________________________________________________________________________________ 
Algorithm 2 Determining the minimal sum of disparities U∗(g) of a cluster g 
Require: A node of a dendrogram 
Ensure: The minimal possible sum of disparities for this node 
  1:  if is.leaf(node) then 
 2:       return (R − 1)/R 
 3:  else 
 4:       return min{U(node),Recall(node.left) + Recall(node.right)} 
 5:  end if______________________________________________________________________________    

For a node in the dendrogram, we denote by *U*(node) the disparity of the corresponding cluster and by *U*^∗^(node) the minimal sum of disparities of the dendrogram induced by (or below) this node. Algorithm 3 can now be used to find the best set of clusters. A cluster is added to the list of final clusters, if its disparity is lower than every sum of disparities obtained when further splitting this node.

 
__________________________________________________________________________________________ 
Algorithm 3 Finding the optimal set of clusters G∗___________________ 
Require: A dendrogram with a root 
Ensure: A list correponding to the optimal set of clusters G∗, obtained by local 
     pruning of the dendrogram node = root 
  1:  if U(node) == U∗(node) then 
 2:       Add all objects belonging to the cluster corresponding to node as one 
     cluster to list 
  3:  else 
 4:       Recall(node.left) 
  5:       Recall(node.right) 
  6:  end if 
 7:  return list____________________________________________________________________________________    

#### Measuring similarity with aggregated disparities

Finally, we can calculate the similarity of a set of LDA runs using the optimized set of clusters. We normalize the sum of disparities of the optimal clustering, such that its values lie in the interval [0, 1], where 0 corresponds to the worst case and 1 to the best case. The worst case is a pruning state with *R* clusters, each consisting of all topics from one LDA run. Then the pruning of Algorithm 3 would lead to a set $\tilde {G}$ of *N* single topic clusters, resulting in the highest possible value for the sum of disparities

(6) \begin{eqnarray*}{U}_{\Sigma ,\mathbf{max}}:=\sum _{g\in \tilde {G}}U(g)=N\cdot \frac{R-1}{R} .\end{eqnarray*}

The similarity measure S-CLOP (Similarity of multiple sets by Clustering with LOcal Pruning) then is defined by

(7) \begin{eqnarray*}\text{S-CLOP}(G):=1- \frac{1}{{U}_{\Sigma ,\mathbf{max}}} \sum _{g\in G}U(g)\in [0,1]\end{eqnarray*}

and S-CLOP(*G*^∗^) = max_*g*∈*G*_S-CLOP(*G*) defines the similarity of replicated LDA runs based on the identified optimal set of clusters *G*^∗^.

### Using S-CLOP for LDAPrototype

As described in ‘LDAPrototype: a new selection algorithm for LDA models’, the LDAPrototype algorithm (cf. Algorithm 1 ) relies on finding the medoid using a suitable similarity measure for LDA models. For this purpose, we use the S-CLOP measure from ‘S-CLOP: a new similarity measure for LDA models’. In terms of the LDAPrototype selection procedure, to compute the S-CLOP similarities we always compare only two LDA runs, so that the clustering and the measuring of disparities aims at matching pairs of topics from the two different runs. Note that in this special case of comparing just two LDA runs with the same number of topics *K*, the normalization factor is ${U}_{\Sigma ,\mathbf{max}}=2\cdot K\cdot \frac{1}{2} =K$. With respect to the definition of *U* we can simplify Eq. (7) to get

(8) \begin{eqnarray*}\text{S-CLOP}(G)=1- \frac{1}{2K} \sum _{g\in G}{|}g{|} \left( {|}{|}{g}_{{|}1}{|}-1{|}+{|}{|}{g}_{{|}2}{|}-1{|} \right) \in [0,1],\end{eqnarray*}

where *g*_|1_ and *g*_|2_ denote groups of *g* restricted to topics of the corresponding LDA run. By using the described pruning algorithm (cf. ‘S-CLOP: a new similarity measure for LDA models’) in the two-LDA case, we obtain a set consisting of groups of size two or one. This means that a topic either has a directly similar counterpart topic in the other LDA, or that it forms a cluster itself. A topic always forms a cluster itself if it is either most similar to another topic from the same LDA, or if the complete linkage distance to one of the already found clusters of matched topics is smaller than to all remaining single topics of the other LDA run. Then, the medoid is determined by the run that maximizes the average pairwise S-CLOP value to all other LDA runs from the same set, namely $\bar {{s}_{i}},$i =1 , …, *R* in Fig. 1 and in Algorithm 1 .

## Data

In ‘Results’ we consider six different datasets, three of which are freely available through R packages (R Core Team, 2020). Table 2 gives an overview of the datasets. Among them are three corpora consisting of traditional newspaper articles. The dataset *reuters* contains 91 articles from 1987 and is included in the package ldaPrototype (Rieger, 2020). The datasets *usatoday* and *nyt* are available to us *via* the paid service of LexisNexis (2019), with more than 7,000 articles and almost two million texts, respectively. They offer a good possibility to test the method on datasets of common and large size. For the latter, we consider all articles from the New York Times from 01/01/1999 to 12/31/2019. From the R package tosca (Koppers et al., 2020), we also use the *economy* and *politics* datasets, which consist of nearly 2,000 and just over 4,000 Wikinews articles from 2004–2018 and 2004–2009, respectively. Also, in contrast to the other datasets, we consider a collection of nearly four million German-language tweets from March 19–June 27, 2020, the first 101 days after then-German Chancellor Merkel’s TV address on the coronavirus outbreak. For this purpose, 50,000 tweets with keywords related to the coronavirus were scraped every hour over the mentioned period using the Twitter API and duplicates were removed (Rieger & Von Nordheim, 2021).

**Table 2:** Specifications of the six considered datasets.

Dataset	Type	Time	M	V (Limit)	K	Source
reuters	Newspaper	1987	91	2,141	5–15	Rieger (2020)
economy	Wikinews	2004–2018	1,855	7,099(5)	20	Koppers et al. (2020)
politics	Wikinews	2004–2009	4,178	12,138(5)	30	Koppers et al. (2020)
usatoday	Newspaper	06-11/2016	7,453	25,486(5)	50	LexisNexis (2019)
tweets	Twitter	03-06/2020	3,706,740	17,208(250)	25	Rieger & Von Nordheim (2021)
nyt	Newspaper	1999–2019	1,993,182	74,218(250)	100	LexisNexis (2019)

All six corpora are preprocessed in R with the packages tosca and tm (Feinerer, Hornik & Meyer, 2008), using common procedures in natural language processing (NLP). That is, duplicates from articles that occur more than once are removed, so that every unique article remains once. As an example, 204 articles were removed from the *usatoday* dataset, which previously contained 7,657 articles. As common in practice, characters are formatted to lowercase; numbers and punctuation are removed. In addition, a trusted stopword list (Feinerer, Hornik & Meyer, 2008) is applied to remove words that do not help in classifying texts in topics. Moreover, the texts are tokenized and words with a total count less or equal to a given limit are neglected. For example, for the *usatoday* dataset we choose this limit to be 5. This reduces the vocabulary size from 79,734 to *V* = 25,486. For the larger datasets *tweets* and *nyt* we have set the limit higher to 250.

For all corpora, we heuristically choose a reasonable number of topics to model. We choose a higher number for larger and more general datasets. As can be seen in ‘Comparison of different values for the parameters R and K’, for the *reuters* dataset we try different numbers of topics, namely *K* = 5, …, 15, and investigate them with respect to the effects on modeling and runtime behavior. Other well known and widely used packages for preprocessing and/or modeling of text data are quanteda (Benoit et al., 2018), topicmodels (Grün & Hornik, 2011) and stm (Roberts, Stewart & Tingley, 2019).

## Study Design

In the following section, we apply the previously defined methods on the six presented datasets in different comparisons. For this purpose, the statistical programming software R version 4.0.2 (R Core Team, 2020) and in particular the package ldaPrototype are used. This is based on an effective implementation in C/C++ of the LDA from the package lda (Chang, 2015). For computation on a batch system or local parallelization, we use the packages batchtools (Lang, Bischl & Surmann, 2017) or parallelMap (Bischl, Lang & Schratz, 2020). For modeling we always use the default parameters unless otherwise specified. For the number of topics *K* to be modeled, the methods deliberately do not provide a default. Our chosen parameters depending on the dataset are given in Table 2. The parameters *α* and *η* are chosen by default as alpha =eta =1/*K*, the Gibbs sampler runs for num.iterations = 200 iterations each time. For the computation using the thresholded version of the Jaccard coefficient tJacc defined in Eq. (3), we choose limit.abs = 10, atLeast = 0, and, unless otherwise specified, limit.rel = 0.002. In addition, *R* = 100 runs of LDAs are modeled by default.

**Figure 2:**
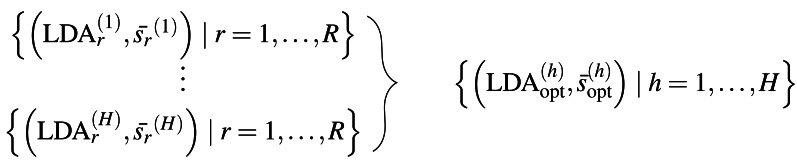
Sets of tuples consisting of LDA models and their mean LDA similarity values to all other LDAs from the same set. Schematic representation of the repetition strategy with *H* repetitions of the LDAPrototype method, each based on *R* basic LDAs. The *H* repetitions result in *H* prototypes (on the right), which in turn can be compared by pairwise model similarities.

The general procedure is as follows: We select a prototypical LDA from a set of *R* LDAs using the presented method LDAPrototype. We repeat this procedure *H* times. Thus, we obtain the pairwise S-CLOP values of all combinations of *R* LDAs, *H* times each. The pairwise similarity of the *H* most representative LDAs, each selected from *R* LDAs, can then be evaluated using the S-CLOP measure. Then, the distributions of mean similarities of the simple replications ${\bar {{s}_{r}}}^{(h)},$r =1 , …, *R*, *h* =1 , …, *H* are compared to the distribution of mean similarities of the prototypical LDAs ${\bar {s}}_{\text{opt}}^{(h)}$. Figure 2 gives a representation of the sets of tuples consisting of LDAs and their mean similarity to the other LDAs from the same set. A location shift between the distributions represents an increase in reliability due to the use of the selection mechanism. We compute the reliability score *rs* for a set of LDAs based on their mean similarities $\bar {{s}_{1}},\ldots ,\bar {{s}_{L}}$ for a *L* that is equal to *R* or *H* as the arithmetic mean of the similarities (cf. Eq. (1)), which can visually be interpreted as the area between *x* = 0 and the empirical cumulative distribution function (ecdf), naturally bounded on the *y*-axis by 0 and 1. Then, we are able to quantify the gain in reliability for a specific selection criterion comparing the reliability scores $rs({\bar {{s}_{r}}}^{(h)}\mid r=1,\ldots ,R)$ for *h* = 1, …, *H* and $rs({\bar {s}}_{\text{opt}}^{(h)}\mid h=1,\ldots ,H)$. For a better (visual) comparability of ecdfs and scores, we use *H* = *R* = 100 in the following, unless otherwise stated.

**Figure 3:**
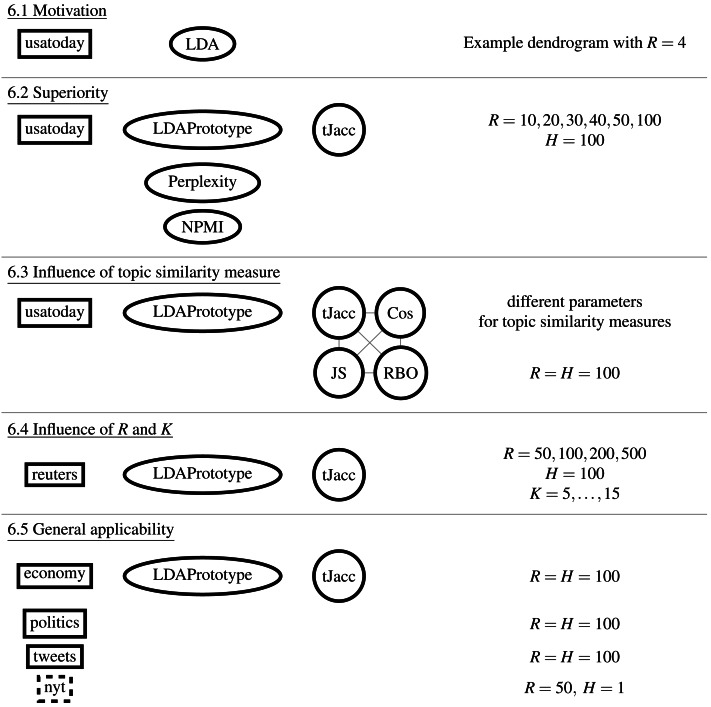
Study overview: Illustration of the datasets, comparative methods, topic similarity measures, and parameters compared.

Figure 3 shows a schematic diagram of the study design. The order corresponds to the subsections of ‘Results’. In ‘Cluster analysis and similarity calculation’, we first show a minimal example of the application of the S-CLOP measure to *R* = 4 runs. In doing so, we analyze the clustering behavior of the topics and exemplify the instability and thus limited reproducibility and reliability of the LDA results. We then show in ‘Increase of reliability’ the improvement in reliability on the same dataset (*usatoday*) in dependence of the parameter *R*, the number of candidate LDAs. In addition, we show that LDAPrototype outperforms perplexity and NPMI regarding the gain in reliability measured by *rs* defined in Eq. (1). This is followed by a comparison of the use of the presented Jaccard coefficient tJacc in Eq. (3) with the other similarity measures (cf. ‘Additionally implemented similarity measures for topics’) cosine (Eq. (15)), Jensen–Shannon (Eq. (14)) and Rank Biased Overlap (Eq. (16)), in ‘Comparison of the implemented similarity measures’. For this, we also use the *usatoday* dataset and also compare the similarity measures in terms of their runtime and parallelizability. Then in ‘Comparison of different values for the parameters R and K’, on a smaller dataset (*reuters*), we compare the runtime and reliability gains for different numbers of topics *K* and different numbers of modeled LDA runs *R*. Finally, we analyze all six datasets in ‘Comparison of the introduced datasets’ and show that the method yields an increase in reliability regardless of the dataset and at the same time remains computable for large datasets.

## Results

Table 3 shows the runtimes for determining the LDAPrototype depending on the different datasets. Due to the size of the *nyt* dataset, only one prototype was determined, and only on *R* = 50 modeled LDAs. In addition, the calculation was not parallelized due to the required memory, so that it lasts around 130 days. For all other datasets *R* × *H* =100 ×100 LDAs, and thus *H* = 100 LDAPrototypes were calculated. The runtimes are observed under parallelization on 4 cores and range from less than a minute for the *reuters* dataset to just over a day for the *tweets* dataset.

**Table 3:** Runtimes for determining the LDAPrototype on the six different datasets.

Dataset	M	R	K	Cores	Min.	Mean	Max.	Unit
reuters	91	100	5	4	43.38	43.81	45.30	secs
economy	1,855	100	20	4	8.25	8.33	8.69	mins
politics	4,178	100	30	4	27.21	27.32	27.76	mins
usatoday	7,453	100	50	4	3.33	3.42	3.56	hours
tweets	3,706,740	100	25	4	28.15	28.64	30.67	hours
nyt	1,993,182	50	100	1	–	130	–	days

**Figure 4:**
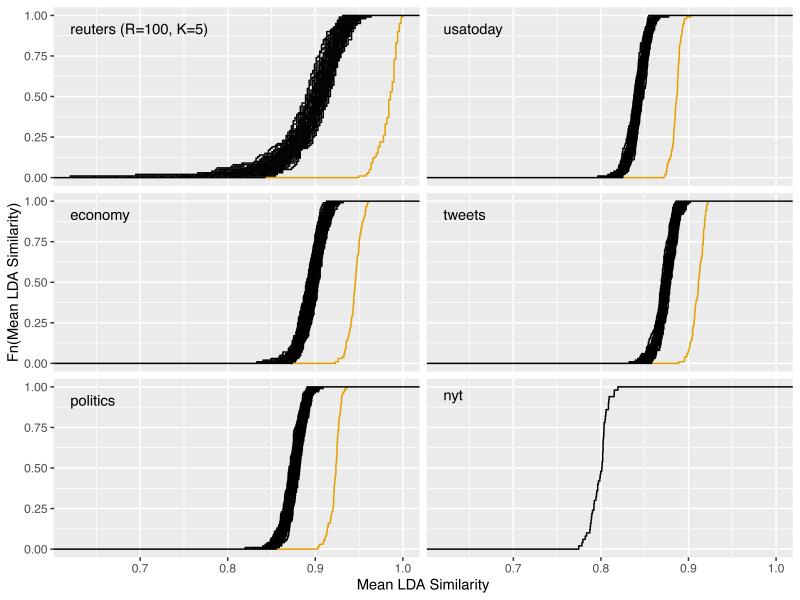
Increase of reliability for five of the six different datasets and reliability of a single LDAPrototype for the *nyt* dataset; black: empirical cumulative distribution functions (ecdf) of the mean pairwise LDA similarites, orange: ecdf of the mean pairwise LDAPrototype similarities.

In Fig. 4, the reliability gain of results from the LDAPrototype method can be seen in comparison to the reliability of basic LDA replications. The (orange) curves, indicating the ecdfs of the mean pairwise similarities of the LDAProtoypes, differ significantly from the (black) ecdfs corresponding to the mean pairwise similarities of the simple LDA replications.

### Cluster analysis and similarity calculation

In this section, we present an example analysis of a clustering result of four independent LDA runs based on newspaper articles from USA Today. We run the basic LDA four times, such that the number of runs to be compared is *R* = 4 and the total number of topics to be clustered is *N* = *R*⋅*K* = 4⋅50 = 200. To demonstrate how dissimilar replicated LDA runs can be, we cluster the *N* = 200 topics from the *R* = 4 independent runs with *K* = 50 topics each using the tJacc coefficient from Eq. (3), complete linkage and the new introduced algorithm for pruning. The four runs were selected from 10,000 total runs. In fact, the runs *Run1* and *Run2* were chosen as the top two models in mean similarity of the 100 prototypes, which means their points lie at the top of the very right (orange) curve in the plot from *usatoday* in Fig. 4. Their similarity values are 0.902 and 0.898 in the set of prototypes or 0.877 and 0.871 in the original sets, respectively. The model *Run3* was chosen as the worst of the 100 prototype models with a similarity value of 0.872 in the set of prototypes and 0.863 in its original set. *Run4* was chosen randomly as one of the worst models realizing a mean similarity to all other models in its original set of 0.807.

We apply hierarchical clustering with complete linkage to the 200 topics. The topics are labeled with meaningful titles (words or phrases). These labels were obtained by hand, based on the ranked list of the 20 most important words per topic. For this, the importance of a word *v* = 1, …, *V* in topic *k* = 1, …, *K* (Chang, 2015) is calculated by

(9) \begin{eqnarray*}I(v,k)= \frac{{n}_{k}^{(\bullet v)}}{{n}_{k}^{(\bullet \bullet )}} \left[ \log \nolimits \left( \frac{{n}_{k}^{(\bullet v)}}{{n}_{k}^{(\bullet \bullet )}} + \right) - \frac{1}{K} \sum _{l=1}^{K}\log \nolimits \left( \frac{{n}_{l}^{(\bullet v)}}{{n}_{l}^{(\bullet \bullet )}} + \right) \right] ,\end{eqnarray*}

where *ɛ* is a small constant value which ensures numerical computability, which we choose as *ɛ* = 10^−5^. The importance measure is intuitive, because it gives high scores to words which occur often in the present topic, but less often in average in all other topics.

**Figure 5:**
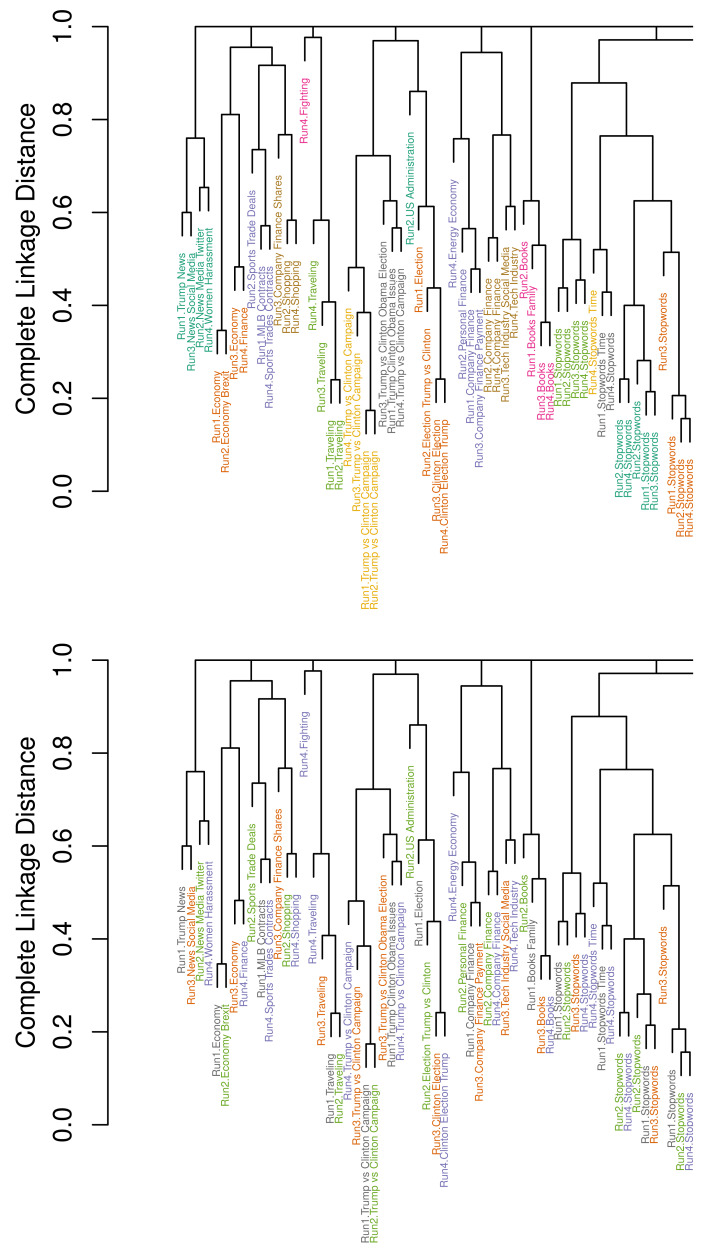
Detail of dendrograms displaying 59 of the *N* = 200 topics from *R* = 4 selected LDA runs on the *usatoday* dataset with *K* = 50 topics each; bottom: colored by runs; top: colored by cluster membership. Complete dendrogram in Fig. B.1.

Figure 5 shows a snapshot of the two dendrograms visualizing the result of clustering the 200 topics and of Algorithm 3 for clustering with local pruning. The vertical axes describe the complete linkage distance based on our tJacc coefficient with limit.rel = 0.002. In the lower dendrogram, the topic labels are colored with respect to the LDA run (*Run1*: grey, *Run2*: green, *Run3*: orange, *Run4*: purple). In the upper dendrogram, topic labels are colored according to the clusters obtained with our proposed pruning algorithm. In addition, every topic label is prefixed by its run number.

Looking at the upper dendrogram, we see that there are a number of clusters with identical labels for topics. This demonstrates that the four LDA runs produce a number of similar topics that are represented by similar word distributions. Examples for such stable topics are *Trump *vs* Clinton Campaign* colored yellow and *Olympics Medals* colored green.

However, there are also considerable differences visible. In the lower dendrogram in Fig. 5, strikingly, there are several topics from *Run4*, highlighted in red, where no other topic is within a small distance. It is remarkable that *Run4* creates such a great number of individual topics, *e.g.*, *Video Games*, *Gender Debate*, *TV Sports* (which includes words for describing television schedules of sport events) and *Terrorism*. Also, *Run4* leads to six explicit stopword topics, which is the maximum number compared to the other runs with four to six stopword topics.

In the upper dendrogram, the color depends on cluster membership. We measure combined stability of these four LDAs by applying the proposed pruning algorithm ( Algorithm 3 ) to the dendrogram. This leads to 61 clusters and a S-CLOP score of 0.83. The normalization factor is given by *U*_Σ,**max**_ = *K*⋅(*R* − 1) = 50⋅3 = 150, and the minimization of the sum of disparities yields *U*^∗^ = *U*_Σ_(*G*^∗^) = 25, resulting in S-CLOP = 1 − 25/150 = 0.83. There are seven single topics, one from each of the first three runs and four from *Run4*. The eleven clusters, which consist of exactly three topics, contain ten times a topic from *Run1*. Topics from *Run2* and *Run3* are represented nine times each, whereas only five of the mentioned clusters contain a topic from *Run4*. This shows that LDA run *Run4* strongly differs from the others. There are a lot of cases, where only one topic from this run is missing to obtain perfect topic clusters with exactly one topic from each run.

For comparison to the proposed local pruning algorithm, we once applied an established global criterion. Since 50 topics were originally modeled for each run, it is not reasonable to determine less than 50 clusters. Therefore, we try the global criterion with 50, …, 70 as the target cluster count. The largest similarity value according to S-CLOP based on the resulting clusters is obtained as 0.3 for 59 clusters. However, considering the dendrogram, we do not observe such large differences in the topic structures between the runs to justify such a low similarity. This shows the necessity of the presented local pruning method.

In addition, the dendrograms illustrate that random selection can lead to a poor model regarding interpretability and especially to some kind of an outlier model as *Run4*. This means that random selection can indeed lead to low reliability.

### Increase of reliability

As an improvement for LDA result selection, we recommend to use the introduced approach based on prototyping of replications. It increases mean similarity, which comes along with an increase in reliability. We demonstrate how to determine a prototype LDA run as the most representative run out of a set of runs, based on our novel pruning algorithm. We show that this technique leads to systematically higher LDA similarities, which suggests a higher reliability of LDA findings from such a prototype run. This is indeed the case in comparison to basic LDA replications, but also in comparison to the well known selection criteria perplexity and NPMI. We calculate perplexity (Grün & Hornik, 2011) by

(10) \begin{eqnarray*}\exp \nolimits \left\{ - \frac{\sum _{m=1}^{M}\sum _{v=1}^{V}{n}_{\bullet }^{(mv)}\log \nolimits \left( \sum _{k=1}^{K}{\hat {\theta }}_{m,k}{\hat {\phi }}_{k,v} \right) }{\sum _{m=1}^{M}{N}^{(m)}} \right\} ,{n}_{\bullet }^{(mv)}=\sum _{k=1}^{K}{n}_{k}^{(mv)}\end{eqnarray*}

and NPMI with respect to the definition from Röder, Both & Hinneburg (2015). The authors offer a web service (see https://github.com/dice-group/Palmetto) to retrieve NPMI scores using the English Wikipedia as reference corpus. The score indicates a normalized pointwise mutual information for one topic using co-occurrences of the first 10 top words of the topic, determined using the score in Eq. (9). The NPMI score for one LDA model is then defined as the mean NPMI scores of all topics from the model.

The introduced similarity measure S-CLOP Eq. (7) quantifies pairwise similarity of two LDA runs by

\begin{eqnarray*}1- \frac{1}{50} \sum _{g\in {G}^{\ast }}U(g)=1- \frac{1}{100} \sum _{g\in {G}^{\ast }}{|}g{|} \left( {|}{|}{g}_{{|}1}{|}-1{|}+{|}{|}{g}_{{|}2}{|}-1{|} \right) , \end{eqnarray*}

where *K* = 50 is the number of topics per model and *G*^∗^ an optimized set of topic clusters identified by our proposed pruning algorithm. We investigate the mean S-CLOP scores per LDA on the corpus from USA Today newspaper articles.

**Figure 6:**
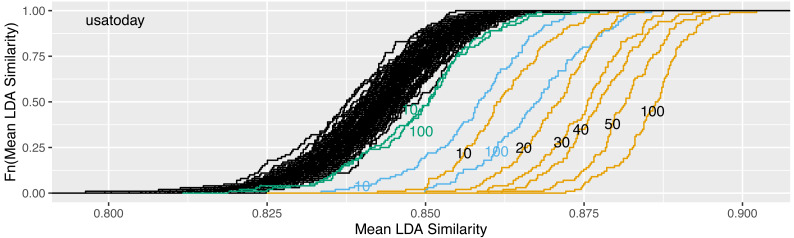
Increase of reliability in dependence of the number of replications *R* on the *usatoday* dataset: ecdfs of the mean similarities calculated on *R* = 100 replications of randomly selected LDA runs (black) and on the *H* = 100 most representative prototype LDA runs (orange) based on subsamples of *R* = 10, 20, 30, 40, 50 or all 100 LDA runs. For comparison, the ecdfs of the 100 selected LDA runs using perplexity (blue) and NPMI (green) based on the subsamples of 10 runs and all 100 runs are given.

We propose to select the LDA run with highest mean pairwise similarity to all other runs. The following study shows that this is a suitable way to identify a stable prototype LDA, thus leading to improved reliability of LDA findings based on this particular run. We fit *R* = 100 LDA models and select the model with highest mean similarity as prototype. This procedure is repeated *H* = 100 times, which results in 100 prototype models. Then, for the 100 prototypes, also mean pairwise similarities to the other prototypes are calculated according to the schema in Fig. 2. The results are visualized in Fig. 6.

The very right curve describes the empirical cumulative distribution function of the mean similarities obtained for the 100 prototypes. At the very left there are the *H* = 100 curves of the 100 × 100 original runs. In addition, we also determine 100 prototypes from subsamples. For this, only 10, 20, 30, 40 or 50 runs from each original set of *R* = 100 runs are randomly selected and are used for the following calculation steps. The resulting curves are plotted and labeled in Fig. 6, the aggregated reliability scores are given in Table 4.

**Table 4:** Reliability scores according to Fig. 6. Comparison of the minimum, mean, and maximum reliability scores based on the *H* = 100 sets of basic LDA replications and the reliability scores after selection by LDAPrototype, perplexity, or NPMI in dependence of the number *R* of candidate models used. Highest (best) value per column in bold.

*R*	10	20	30	40	50	100
Replications						
Min.	0.8338	0.8356	0.8378	0.8368	0.8359	0.8374
Mean	0.8429	0.8428	0.8426	0.8425	0.8425	0.8425
Max.	0.8515	0.8496	0.8483	0.8490	0.8487	0.8477
Selection						
LDAPrototype	**0.8622**	**0.8703**	**0.8750**	**0.8774**	**0.8816**	**0.8858**
Perplexity	0.8578	0.8612	0.8629	0.8629	0.8655	0.8674
NPMI	0.8485	0.8502	0.8519	0.8502	0.8493	0.8493

The minimum of the mean similarities from the original 100 sets of 100 models is 0.796, while the maximum is 0.877. It turns out that NPMI does not show a good ability to increase the reliability of LDA runs. For the reliability score of NPMI-selected runs, it makes little difference whether 10 or 100 models are available to the selection procedure. This suggests that the measure is better suited for other optimization goals than for increasing reliability. The perplexity does a relatively good job in the task of improving the reliability. For *R* = 100 candidate models, the relative reduction in the remaining improvement to the maximum reliability of 1 in comparison to random selection is 38% for our method LDAPrototype, namely (1 − 0.8425)/(1 − 0.8858) = 0.1575/0.1142 = 1.38, and 19% (0.1575/0.1326) for selection by perplexity. Therefore, the improvement in reliability for *R* = 100 is half that achieved by LDAPrototype. Overall, it can be seen that our method outperforms the other two popular and well-known selection methods in terms of increasing reliability.

Based on the findings from Fig. 6 and Table 4, we recommend to fit at least 50 replications because this leads to an increase of similarity to 0.862 at the minimum and 0.895 at the maximum, or a reliability score (mean) of 0.8816, respectively. This corresponds to a relative reduction of the remaining possible improvement of 33%. Higher values for the number of repetitions are desirable. In general, the choice depends on the complexity of the corpus. Encapsulated topics or certain complicated dependency structures make the modeling procedure more prone to a larger span of possible fits and therefore to smaller mean similarity values. However, if computational power is limited, already taking the prototype model from 10 candidates considerably increases the reliability. Here, the minimum and maximum of mean similarity are 0.842 and 0.880 (*rs* = 0.8622), considerably higher values than these associated with random selection and 14% relative reduction of remaining improvement.

### Comparison of the implemented similarity measures

Up to this point, we have performed all calculations with our tJacc Eq. (3) in ‘Thresholded version of the Jaccard coefficient: a similarity measure for topics’. Now we study differences in reliability with different choices of measures of topic similarity. For this we compare cosine similarity Eq. (15), Jensen–Shannon similarity Eq. (14) and Rank Biased Overlap (RBO) Eq. (16) with the tJacc coefficient. We consider, where applicable, effects of different parameter constellations on the correlation of the similarities. We show that the tJacc coefficient is a good choice for the topic similarity measure, because it improves reliability while not having a disproportionate runtime.

**Figure 7:**
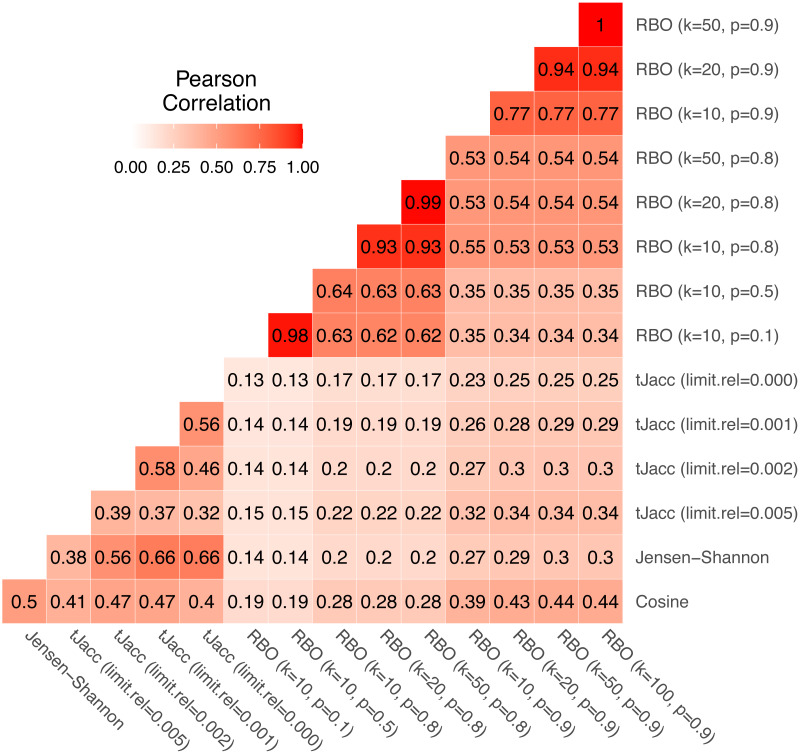
Correlation matrix of pairwise LDA similarity values calculated using the four similarity measures with selected parameter combinations for 5,000 topics on the *usatoday* dataset.

We again consider the *usatoday* dataset for the comparison. For the tJacc coefficient we set limit.abs = 10 and atLeast = 0 and vary limit.rel ∈{0, 0.001, 0.002, 0.005}, for the RBO we choose eight combinations of *k* ∈ {10, 20, 50} and *p* ∈ {0.1, 0.5, 0.8, 0.9}. We compute all pairwise topic similarities of the total *R*⋅*K* = 5000 topics using the given measures and first consider the correlation of the pairwise LDA similarities based on these. In Fig. 7 all pairwise correlations of the similarity values are given.

Thereby, clear patterns can be identified. The similarities obtained with cosine and Jensen–Shannon are correlated with a value of 0.50. The similarity values based on the tJacc coefficient correlate with the cosine similarity depending on the parameter in the range from 0.40 to 0.47. It is noticeable that the correlation initially increases from 0.41 to 0.47 when considering a longer tail of words belonging to a topic, but drops to 0.40 when considering the complete tail. In contrast, the correlation with Jensen–Shannon LDA similarities increases steadily from 0.38 for limit.rel = 0.005 to 0.66 when considering the complete tail, or these words that were assigned to that topic at least 11 times (limit.abs = 10). It is interesting to note that even the different versions of the tJacc coefficient are mostly less correlated with each other than the tJacc LDA similarities are with the Jensen–Shannon LDA similarities. This illustrates that there can be significant differences between different choices for the threshold based on limit.rel.

As mentioned in ‘Thresholded version of the Jaccard coefficient: a similarity measure for topics’, choosing the parameter limit.rel as 0.002 determines around 100 words per topic as relevant. Thus, one could assume that the corresponding similarities should correlate strongly with those of the RBO with *k* = 100. In fact, we see that the RBO is rather weakly correlated with the other three measures overall; in particular, for low values of *k* ≤ 50 and *p* ≤ 0.8. The corresponding correlations are all below 0.30. However, it is also noticeable that for increasing *k* and *p*, the RBO similarities appear to be increasingly correlated with the other three similarity measures. This is due to the fact that for larger values of *k* and *p* the RBO converges to a measure very similar to the Jaccard coefficient or for *p* ≠ 1 to the AverageJaccard defined in Eq. (11) in Appendix A. This means that RBO similarities calculated with parameters *k* = 100 and *p* = 1 should be very close to tJacc similarities with limit.rel = 0.002. In the present case, however, even with 0.9, *p* is still much smaller than 1. Note that 0.9^100^ ≈ 0 and thus the 100th word in the ranked list gets practically no weight, which is exactly the idea of the RBO. Therefore it is not recommended to choose the parameter *p* close to 1. Alternatively, a better approach would be to choose an implementation based on Jaccard, *e.g.*, AverageJaccard, because it is faster to implement. These findings show that *k* and *p* should always be chosen dependent on each other. One can also see in Fig. 7 that words from rank 50 onwards no longer have a significant influence with a choice of *p* = 0.9. The correlation between the LDA similarities based on RBO with *k* = 50, *p* = 0.9 and *k* = 100, *p* = 0.9 is 1.

The four measures considered have different complexities in terms of their implementations. While the cosine similarity can be computed very quickly, the tJacc coefficient and the Jensen–Shannon similarity require computationally intensive precomputations, which increases the runtime. Cosine similarity and Jensen–Shannon similarity have no parameters to be set. For the tJacc coefficient, the calculations do not depend on the chosen parameters. The calculation of the RBO is generally time-consuming, since values must be calculated individually for all considered depths up to the maximum rank, which also means that the runtime for the calculation of the RBO strongly depends on the parameter *k*.

The runtimes are documented in Table 5. The times here are in hours, the calculations were run on four cores in parallel and are based on at least 100 different values. For each measure, a serial calculation was performed once in each case in order to be able to give a value for estimating the parallelizability of the calculations or implementations. It can be seen that the cosine similarity is the fastest to calculate with well under one hour. The thresholded Jaccard coefficient requires slightly more than twice as much time, while the Jensen–Shannon similarity computes with 83 and 275 min, respectively, again almost twice as long as the tJacc coefficient. The Rank Biased Overlap with *k* = 100 takes even over 2 days in parallel computation. This is also due to the fact that the implementation is only parallelized with a score of 0.6, *i.e.,* one achieves only 60% of the maximum possible time saving through parallelization. With a maximum score of 1, the calculation would only take 30.97 hours instead of 51.62. The cosine similarity is also not implemented maximally parallelized. This is due to the overall low runtime. The tJacc coefficient, instead, is implemented in parallel with an approximate maximum time saving of 0.93.

**Table 5:** Runtime and parallelizability comparison of the four similarity measures computing pairwise similarities of *R*. *K* = 5000 topics on the *usatoday* dataset; all runtimes refer to hours on 4 cores, a measure is better implemented in parallel for a larger parallelizability score ∈[0.25, 1].

	Measure	Cos	tJacc	JS	RBO	RBO	RBO	RBO
	k	–	–	–	10	20	50	100
Parallel (4 Cores)	Min.	0.26	0.65	1.33	4.54	9.09	22.47	47.15
	Mean	0.29	0.68	1.39	5.05	11.40	24.75	51.62
	Max.	0.31	0.76	1.48	6.07	12.13	29.37	59.05
Serial	Time	0.86	2.49	4.58	–	27.26	–	–
Parallelizability	Score	0.75	0.93	0.83	–	0.60	–	–

After comparing the measures in terms of runtime and correlation of the resulting pairwise S-CLOP values, we restrict the analysis to one parameter combination per measure and compare the increase in reliability in dependence of the measure.

**Figure 8:**
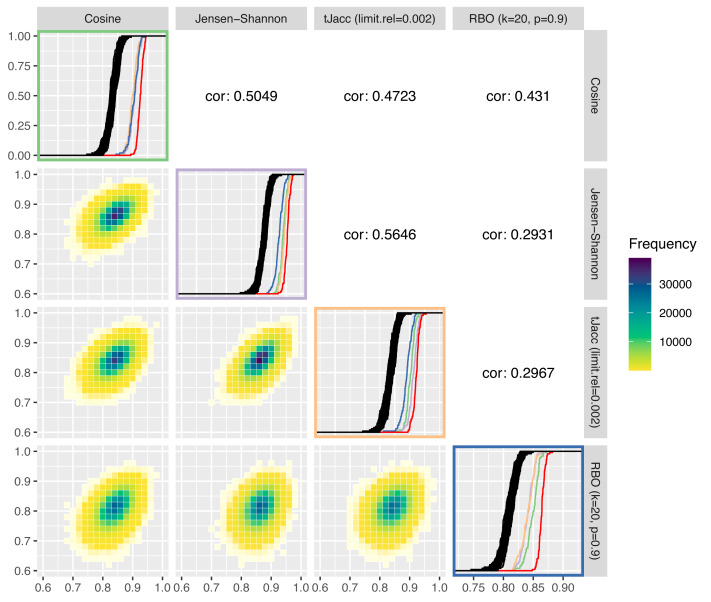
Comparison of four selected similarity measures regarding their increase of the reliability of LDA results: on the diagonal the ecdfs of the LDA similarities based on the different similarity measures are shown in black, in addition the similarities of the LDAPrototypes are shown in red or in further colors for the LDAPrototypes determined on the basis of the other measures (green for cosine, purple for Jensen–Shannon, orange for tJacc and blue for RBO); below the diagonals correlation plots of the LDA similarities according to the similarity measure are shown as heatmap, above the diagonals the corresponding correlations are given.

In Fig. 8, these are plotted against each other. We compare the cosine similarity, Jensen–Shannon similarity, the tJacc coefficient with limit.rel = 0.002 and the RBO with *k* = 20, *p* = 0.9. On the diagonal of the plot matrix, the known ecdfs of the LDA similarities are shown in black and the ecdf of the LDAPrototypes in red, respectively. In addition, we calculated—using the same measure—the similarities of the LDAPrototypes, that were determined based on the other three topic similarities. The colors of the ecdfs correspond to the color of the box of similarity measures. This allows the different measures to be analyzed in terms of their selection behavior taking into account the level differences of the similarity values. Table 6 gives the corresponding reliability scores of the ecdfs on the diagonal. The lower triangular plot matrix provides the LDA similarities as pairwise correlation plots, the upper matrix the corresponding correlations themselves. Determining the pairwise LDA similarities based on 100 experiments with *R* = 100 replications each yields 100⋅(*R* − 1)*R*/2 = 495000 values per similarity measure, on which the heatmaps and correlations are both based.

**Table 6:** Reliability scores according to Fig. 8. Comparison of the topic similarity measures with respect to the increase in reliability. Each column shows the reliability measures depending on the used topic similarity measure, the lower four rows indicate which similarity measure is used for the selection of the *H* = 100 prototypes based on the respective *R* = 100 basic LDAs. Highest value per column in bold.

	Comparison of LDA runs based on			
	Cos	tJacc	JS	RBO
Replications				
Min.	0.8364	0.8374	0.8587	0.8039
Mean	0.8413	0.8425	0.8634	0.8101
Max.	0.8459	0.8477	0.8666	0.8155
LDAPrototype				
Cos	**0.8878**	0.8764	0.8951	0.8487
tJacc	0.8748	**0.8859**	0.8970	0.8403
JS	0.8778	0.8811	**0.9019**	0.8410
RBO	0.8771	0.8700	0.8884	**0.8642**

It can be seen that the LDA similarities according to the tJacc coefficient and according to the Jensen–Shannon similarity are most highly correlated with each other. The point cloud is least circular, but rather narrow and elliptical in shape. It can also be seen that the RBO LDA similarities are very different from the others. In the lower right plot (outlined in blue), the three ecdfs of different colors show significant differences from the red curve, with the cosine curve showing even slightly more similarity than the curves of the other two measures. Similarly, the blue curve is in the Jensen–Shannon (purple) and tJacc (orange) windows far behind the other colored ecdfs. The cosine LDA similarities (top left, green) differ barely for all the foreign-determined LDA prototypes, but the red curve sets itself apart from the others. Thus, the selection by cosine similarity obviously also differs significantly from the others.

The plots from Fig. 8 suggest that all four measures differ with regard to their selection criteria. However, a qualitative ranking which of the measures increases the reliability of the results the most is difficult because for this question the true evaluation measure has to be identified first, which is a vicious circle. For this reason, all these measures have their justification to be used within the procedure. We prefer to use the thresholded Jaccard (tJacc) coefficient because it has plausible heuristics and reasonable runtime. Its selection of the LDAPrototype strongly correlates with that of the Jensen–Shannon similarity, for which, according to Appendix A, besides the tJacc coefficient itself, the best results in terms of correlations with human perceptions could be obtained.

### Comparison of different values for the parameters R and K

In addition to the choice of the similarity measure, the choice of the parameters *K*, number of topics to be modeled, and *R*, number of replications, also has a large influence on the runtime of the method. In ‘Increase of reliability’ it has already been shown that larger values for *R* result in a larger increase in reliability. We will confirm these findings based on the *reuters* dataset on the one hand and on the other hand bring them into a combined comparison with the number of modeled topics.

In Table 7 the runtimes for the determination of the LDAPrototypes based on the calculations of the topic similarities by the tJacc coefficient are given. Obviously, the runtime increases more than linear in both in the number of topics *K* to be modeled and in the number of replications *R*. This is due to the fact that the computation of the matrices of topic similarities have a quadratic complexity, since pairwise similarities are computed. The runtime for modeling the LDAs is linear in the parameters *R* and *K*.

**Table 7:** Runtime comparison of LDAPrototypes on the *reuters* dataset for choices of *R* and *K*; all runtimes refer to minutes on 4 cores.

*R*	50	100	200	500
*K* = 5				
Min.	0.25	0.72	2.46	16.01
Mean	0.26	0.73	2.49	16.31
Max.	0.31	0.76	2.77	17.16
*K* = 10				
Min.	0.47	1.43	5.47	36.45
Mean	0.48	1.47	5.57	37.03
Max.	0.53	1.64	5.84	38.25
*K* = 15				
Min.	0.79	2.57	9.81	69.28
Mean	0.80	2.65	10.03	70.03
Max.	0.82	2.81	10.37	71.58

**Figure 9:**
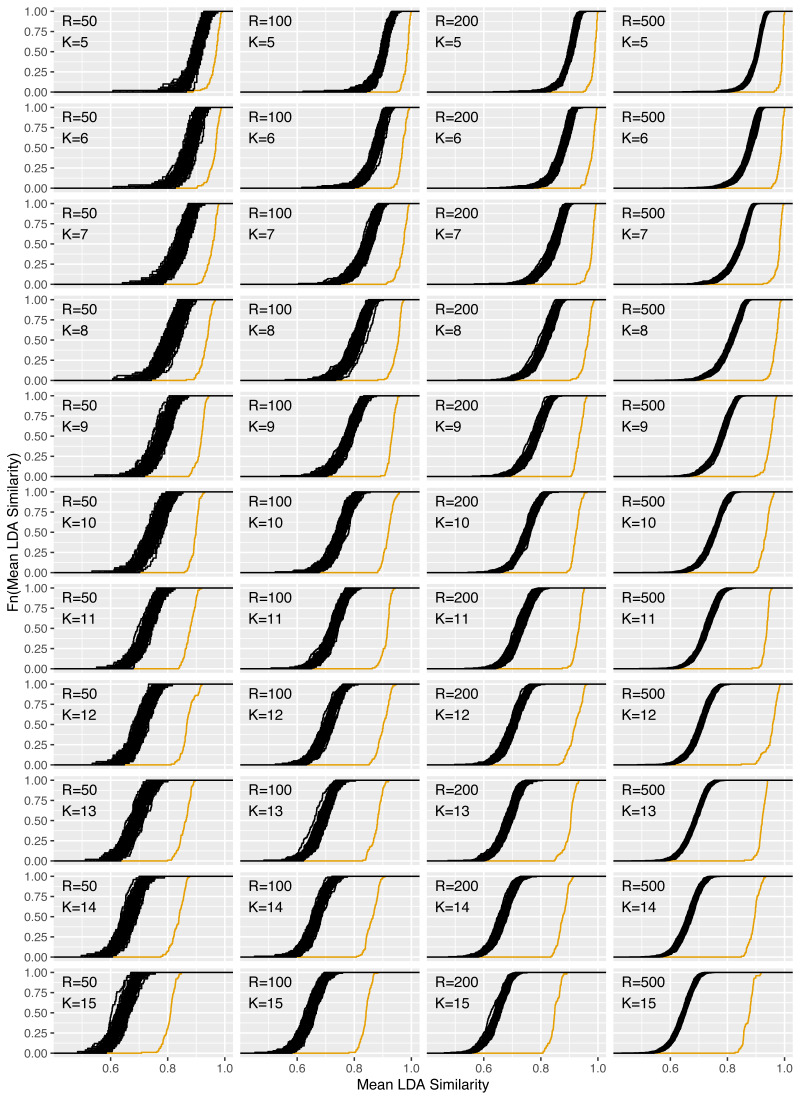
Increase of reliability for the *reuters* dataset for *R* = 50, 100, 200, 500 and *K* = 5, …, 15.

Figure 9 shows the corresponding plots for the increase in reliability for all combinations of *R* = 50, 100, 200, 500 and *K* = 5, …, 15 and Table B.1 in the Appendix the corresponding reliability scores. Consistent with expectations, for fixed *K*, increasing the replication number from 50 to 500 does not change the location parameter for the ecdfs of the mean pairwise LDA similarities. However, the variance of the similarities decreases due to the higher replication number. It can be seen that for fixed *R* and increasing topic number *K* the level of similarities decreases. From an average similarity of 0.90 for *K* = 5 for the LDA replications, the value decreases to 0.75 for *K* = 10 and 0.65 for *K* = 15. The increase in reliability is marked by the orange ecdfs and is clearly pronounced for all parameter combinations. The gain is larger for higher topic numbers, so the level of LDAPrototype similarity does not decrease quite as much with increasing parameter *K*. The gain is also larger for increasing *R*. This is an expected behavior because then each prototype is determined from a larger set of individual LDAs and thus each LDAPrototype becomes even more reliable. For *K* = 5, the similarities of the prototypes thus increase from 0.97 for *R* = 50 to close to 1 for *R* = 500 (with LDA similarities around 0.90). For *K* = 9 they increase from 0.91 to 0.95 (0.78) and for *K* = 14 from 0.84 to 0.90 (0.67).

The findings from Fig. 6 are confirmed here. With increasing *R* the reliability gain increases. However, already for small values of *R* a clear increase is recognizable. Accordingly, *R* should be chosen as large as possible depending on the available computing power.

### Comparison of the introduced datasets

For the corpus of 7,453 newspaper articles from the USA Today from 01/06 to 11/30/2016 and the *reuters* dataset with *M* = 91, an increase in reliability could clearly be shown. Lastly, we show that this increase results independently of the dataset, so that the LDAPrototype method can be reasonably applied to many different types of text data.

**Figure 10:**
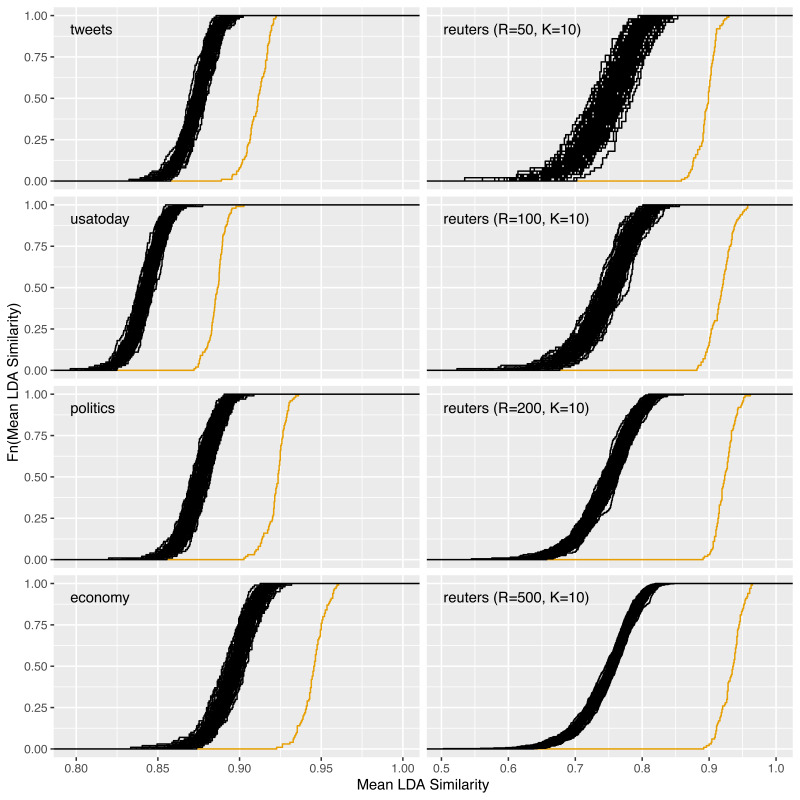
Increase of reliability for the datasets *tweets*, *usatoday*, *politics*, and *economy* with *R* = 100 and for *reuters* with *R* = 50, 100, 200, 500.

**Table 8:** Reliability scores according to Figs. 4 and 10. Comparison of the increase in reliability obtained by using LDAPrototype on the datasets tweets, politics and economy (*R* = *H* = 100). In addition, the reliability score of the basic LDA replications for the nyt dataset is given (*R* = 50).

	tweets	politics	economy	nyt
Replications				
Min.	0.8683	0.8711	0.8885	–
Mean	0.8735	0.8759	0.8963	0.7982
Max.	0.8790	0.8809	0.9029	–
Selection				
LDAPrototype	0.9108	0.9224	0.9453	–

Figure 10 and Table 8 in particular show the increase in reliability for the datasets *tweets*, *politics*, and *economy* that have not yet been considered so far. For comparison, the corresponding plots for the *usatoday* dataset are shown, for *reuters* with *K* = 10 and the four different values for *R* = 50, 100, 200, 500 (cf. ‘Comparison of different values for the parameters R and K’). Figure 4 already showed the computability for large datasets on the example of the *nyt* dataset. We did not repeat the computation of the LDAPrototype 100 times for this dataset due to the comparatively higher runtime. The *tweets* dataset, with 3706740, does consist of a larger number of individual documents than the *nyt* dataset (1,993,182, cf. Table 2). However, due to the more specific topic structure, we chose a smaller topic number for this dataset. Together with the fact that the modeled tweets are much shorter than journalistic texts from the New York Times, this leads to a significantly lower runtime of just over one day per LDAPrototype than for the *nyt* dataset with about 130 days (cf. Table 3).

The plots show a lower gain in reliability for *tweets* than for the dataset *economy*, for example. This might surprise because the similarities of the basic LDA repelications are already somewhat higher for the latter. To be concrete, the similarities increase from about 0.88 to 0.94 for *economy* and from 0.87 to 0.91 for the dataset consisting of tweets. This reduced gain could be due to the shorter texts and the resulting higher uncertainty in the modeling. As a result, the instability of the LDA on this dataset is more pronounced, so that higher reliability increases are only possible with larger values for *R*. On the right side of Fig. 10 the results from the ‘Increase of reliability’ and ‘Comparison of different values for the parameters R and K’ are recapitulated. The increase of reliability increases steadily with an increasing number of LDA replications *R*.

## Discussion

Topic modeling is popular for understanding text data, though the analysis of the reliability of topic models is rarely part of applications. This is caused by plenty of possibilities for measuring stability, but missing strategies for increasing reliability without touching the original fitting procedure.

We have presented a novel method to address the reliability issue caused by the inherent instability of the LDA procedure. For this purpose, we want to improve the reproducibility of the results and we talk about highly reliable results if they can be reproduced very well. The presented method is based on the idea of modeling a set of LDAs and then selecting the best, in this case the most central, model through a selection mechanism. We call this medoid of several LDA runs LDAPrototype. We deliberately choose not the best-fit model according to one of the well-known—mostly likelihood-based—measures (Griffiths & Steyvers, 2004; Grün & Hornik, 2011), but the LDA that agrees most with all other LDAs obtained from the same text data using the same parameter settings. We were able to show that the selection of the LDA using our LDAPrototype method strongly increases the reliability. In comparison, for the datasets under consideration in this paper, the improvement turns out to be about twice as large as that obtained from a selection based on perplexity. Moreover, model selection with the optimal NPMI does not significantly improve the reliability compared to the basic LDAs, regardless of the number of candidate models.

In various analyses, we have shown that the presented method increases the reliability of the results. By applying it to different datasets, we have first shown its feasibility due to the implemented R package and at the same time that it produces the desired increase in reliability irrespective of the dataset. We also investigated the influence of the parameters of the number of modeled topics *K* and number of LDA replications *R* on this increase. Furthermore, we presented differences in the determination of the LDAPrototype based on the presented similarity measures. All four measures under consideration resulted in an increase in reliability. No clear ranking could be obtained. The decision for the thresholded Jaccard (tJacc) coefficient as the default measure is based on the combination of visible increase in reliability, interpretability of the measure, fast implementation, and supporting arguments from studies (Aletras & Stevenson, 2014; Kim & Oh, 2011) regarding correlation with human perception.

One limitation of the method is that the repetition strategy could be computationally demanding on large datasets with a large number of topics. This can be seen in the example computation on the New York Times dataset (nyt). However, the results on larger datasets are naturally more stable due to the large size of the dataset. In fact, the study showed that LDAPrototype basically has a larger effect in terms of increasing the reliability of the results on small datasets. For this reason, we see the main application of the method in scenarios with small to medium sized datasets, where computability - also through parallelization and easy interfacing to high performance clusters - is thus not a concern.

The quality of the results of LDAPrototype in terms of correlation with human perception was not in the scope of the paper, but we plan to investigate it in further studies. For this, a study with human coders is necessary and different models like the basic LDA, LDAPrototype, structural topic model, BERTopic, neural topic models, perplexity or NPMI optimized and potentially other models are evaluated regarding their quality by human coders. For this purpose, we focus on meaningful and distinct topics.

In several application examples—for example in communication studies—our proposed method has already produced well interpretable topics in an automated way (*e.g.*, Von Nordheim, Rieger & Kleinen-von Königslöw, 2021). A side effect from our method is that LDAPrototype prevents a human “selection algorithm” that chooses the LDA model that best supports the hypothesis. The LDAPrototype methodology also forms the basis for a new LDA method RollingLDA (Rieger, Jentsch & Rahnenführer, 2021), which was developed for the construction of time-consistent time series using LDA.

The presented idea of selecting a prototypical LDA from a set of LDA runs can be transferred to other topic models as well. For example, the Structural Topic Model offers not only pre-initialized topics, but also the possibility of random initialization. This causes the issue of limited reliability of interpretations due to the lack of reproducibility of the results. At this point, the reliability may also increase using an analogous procedure to the method LDAPrototype.

## Supplemental Information

10.7717/peerj-cs.2279/supp-1 Supplemental Information 1 The code to reproduce the experiments and all analyses

## Figures and Tables

**Figure B.1:**
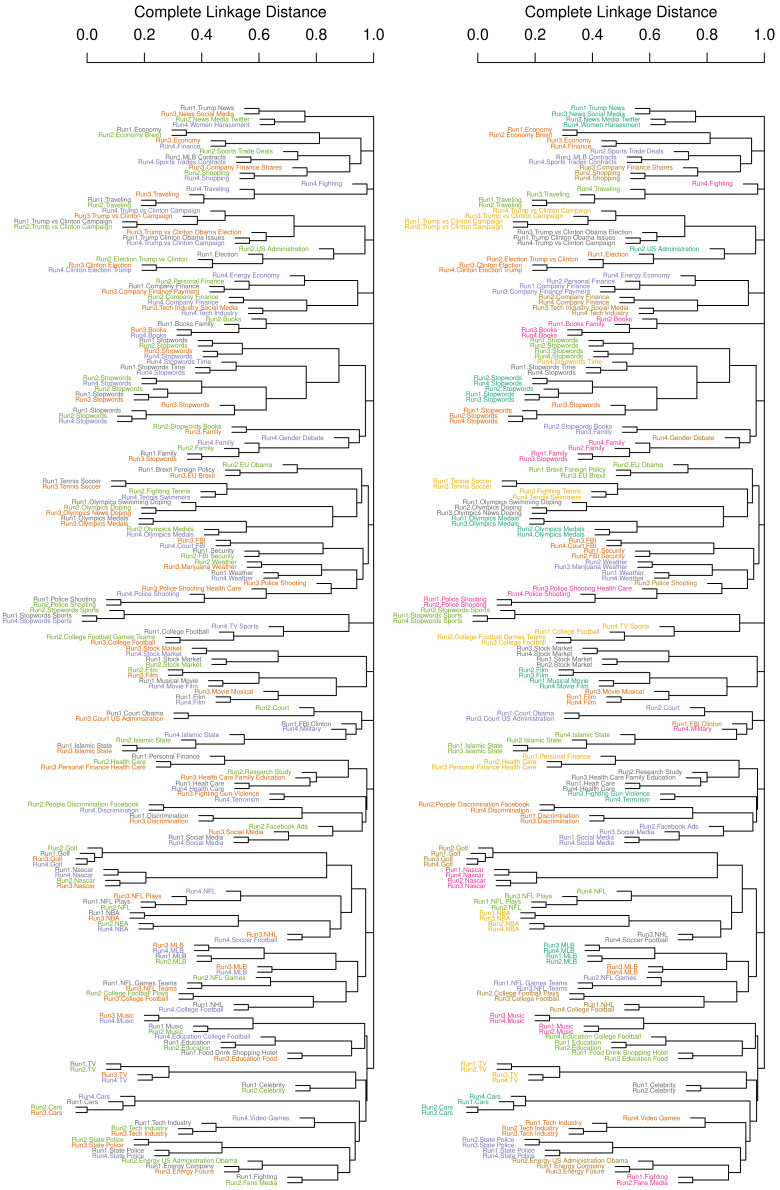
Dendrograms of *N* = 200 topics from *R* = 4 selected LDA runs on the usatoday dataset with *K* = 50 topics each; left: colored by runs; right: colored by cluster membership. Detail can be found in Fig. 5.

## Appendix A. Additionally implemented similarity measures for topics

For our selection procedure LDAPrototype, we choose the tJacc coefficient from ‘Thresholded version of the Jaccard coefficient: a similarity measure for topics’ as main similarity measure for comparing topics based on their word count vectors. In the literature, several alternatives are discussed, from which we compare the three most promising to our thresholded version of the Jaccard coefficient in ‘Comparison of the implemented similarity measures’.

While Agrawal, Fu & Menzies (2018) determine topic similarity with a Jaccard coefficient of the top nine words per topic across multiple runs and measure stability with the median of the topic similarities, Maier et al. (2018) use the cosine similarity (see below). For repetitions of the same modeling procedure they match topics with the highest cosine similarity, which additionally has to be greater than an arbitrarily selected threshold 0.7. Then, for two models the similarity is calculated as the share of topic matches, and for more than two models by the mean of all pairwise shares.

Greene, O’Callaghan & Cunningham (2014) and Su, Greene & Boydell (2016) determine topic similarities with an average Jaccard coefficient

(11) \begin{eqnarray*}\text{AverageJaccard}(A,B)= \frac{1}{N} \sum _{n=1}^{N} \frac{{|}{A}_{n}\cap {B}_{n}{|}}{{|}{A}_{n}\cup {B}_{n}{|}} ,\end{eqnarray*}

where *A*_*n*_ and *B*_*n*_ define the sets of the first *n* words of the ordered lists from the word sets A and B. They choose *N* = 5 and find the best matching topics of different LDA runs based on this measure with the hungarian method (Kuhn, 1955). The authors try to encounter the problem that more than two runs of topics have to be matched by learning a reference model. They calculate the similarity of one LDA to the reference LDA as the mean average Jaccard coefficient over all matched topics, characterized by their word sets *Z*_*k*^∗^_ to the topics ${Z}_{k}^{(\text{ref})}$ of the reference model. Here *Z*_*k*^∗^_ denotes the reordered topics’ word lists *Z*_*k*_ of the LDA run, so that matched topics have the same index. Analogously, they calculate stability over a number of *R* replications as the mean over the pairwise similarities against the topics’ word lists of the (predetermined) reference model ${Z}_{k}^{(\text{ref})}$ by

(12) \begin{eqnarray*} \frac{1}{R} \sum _{r=1}^{R} \left( \frac{1}{K} \sum _{k=1}^{K}\text{AverageJaccard} \left( {Z}_{k}^{(\text{ref})},{Z}_{{k}^{\ast }}^{(r)} \right) \right) .\end{eqnarray*}

One drawback of this approach is the specification of the reference model, which should be a good representative of all other LDAs. It is non-trivial to determine this representative model. Therefore, our approach follows an opposite strategy. We first calculate similarities between models and then determine the prototype model, *i.e.,* the most representative LDA run, based on these values.

Aletras & Stevenson (2014) argue that Jensen–Shannon divergence (Lin, 1991) is one of the best similarity measures based on word distributions considering correlation with human judgments. It is a symmetric version of the Kullback–Leibler divergence (Kullback & Leibler, 1951)

(13) \begin{eqnarray*}\text{KLD} \left( {\mathbi{q}}_{1},{\mathbi{q}}_{2} \right) =\sum _{v=1}^{V}{q}_{1,v}\log \nolimits \frac{{q}_{1,v}}{{q}_{2,v}} ,{\mathbi{q}}_{1},{\mathbi{q}}_{2}\in \left( 0,1 \right. { \left. \right] }^{V}\end{eqnarray*}

and treated as similarity measure defined as

(14) \begin{eqnarray*}\text{JS}({\mathbi{w}}_{i},{\mathbi{w}}_{j})=1- \left( \text{KLD} \left( {\mathbi{p}}_{i}, \frac{{\mathbi{p}}_{i}+{\mathbi{p}}_{j}}{2} \right) +\text{KLD} \left( {\mathbi{p}}_{j}, \frac{{\mathbi{p}}_{i}+{\mathbi{p}}_{j}}{2} \right) \right) /2\nonumber\\\displaystyle =1-\text{KLD}({\mathbi{p}}_{i},{\mathbi{p}}_{i}+{\mathbi{p}}_{j})/2-\text{KLD}({\mathbi{p}}_{j},{\mathbi{p}}_{i}+{\mathbi{p}}_{j})/2-\log \nolimits (2),\nonumber\\\displaystyle {\mathbi{p}}_{l}= \left( {p}_{l,1},\ldots ,{p}_{l,V} \right) = \left( {n}_{l}^{(\bullet 1)},\ldots ,{n}_{l}^{(\bullet V)} \right) /{n}_{l}^{(\bullet \bullet )}.\end{eqnarray*}

Koltcov, Koltsova & Nikolenko (2014) use a normalized variant of the Jensen–Shannon divergence (they refer to it as the Kullback–Leibler divergence) to measure topic stability, and suggest using the measure to identify stable topics in repeated runs. In our study in ‘Comparison of the implemented similarity measures’, we compare the Jensen–Shannon divergence to other approaches for topic similarity measures. Moreover, Aletras & Stevenson (2014) found out that a standard Jaccard coefficient is able to realize higher correlations to human judgments than other common similarity measures on specific datasets.

Kim & Oh (2011) showed that Jaccard coefficients perform on par with Jensen–Shannon divergence and outperform a number of other popular similarity measures like cosine similarity, which is defined as

(15) \begin{eqnarray*}\cos \nolimits ({\mathbi{w}}_{i},{\mathbi{w}}_{j})= \frac{\sum _{v=1}^{V}{n}_{i}^{(\bullet v)}{n}_{j}^{(\bullet v)}}{\sqrt{\sum _{v=1}^{V}{ \left( {n}_{i}^{(\bullet v)} \right) }^{2}}\sqrt{\sum _{v=1}^{V}{ \left( {n}_{j}^{(\bullet v)} \right) }^{2}}} .\end{eqnarray*}

and the mentioned Kullback–Leibler divergence. To quantify the quality of the similarity measures they compare the negative log-likelihood of the model as an indicator how well the model explains the data. They swap the best matching topics from models of two time slices and interpret an increase of the negative log-likelihood as deficiency of the specific similarity measure.

Another option for measuring topic similarity introduced by Mantyla, Claes & Farooq (2018) is the Rank Biased Overlap (RBO) (Webber, Moffat & Zobel, 2010) for comparing ranked lists. The similarity is defined as

(16) \begin{eqnarray*}\text{RBO}(A,B)=2{p}^{k} \frac{ \left\vert {A}_{k}\cap {B}_{k} \right\vert }{ \left\vert {A}_{k} \right\vert + \left\vert {B}_{k} \right\vert } + \frac{1-p}{p} \sum _{d=1}^{k}2{p}^{d} \frac{ \left\vert {A}_{d}\cap {B}_{d} \right\vert }{ \left\vert {A}_{d} \right\vert + \left\vert {B}_{d} \right\vert } \end{eqnarray*}

with parameters *k* ∈ ℕ setting the maximum depth of evaluation and *p* ∈ (0, 1) controlling the influence of higher ranked words. While the measure seems to be useful because it implements a more flexible form of a Jaccard coefficient, the authors do not investigate stability of LDA models based on RBO. Moreover, the calculation of the measure is very time consuming.

For our analysis we prefer the thresholded version of the Jaccard coefficient tJacc as defined in Eq. (3), because in ‘Comparison of the implemented similarity measures’ we show that it performs on par with the others. In addition, it is calculated faster than the Jensen–Shannon divergence and especially than the RBO. In comparison to the cosine similarity, calculation of the tJacc is computationally more demanding, but may lead to an increased interpretability. In view of the large computation demand for the LDA modeling in big data scenarios, this runtime increase is acceptable and rather minor. Our R package ldaPrototype offers the possibility of calculating the prototype based on topic similarities calculated using the cosine, tJacc, Jensen–Shannon or RBO similarity measure.

## Appendix B. ADDITIONAL FIGURES AND TABLES

**Table B.1:** Reliability scores according to Fig. 9. Comparison of different choices of *R* and *K* on the reuters dataset for the increase in reliability obtained by using LDAPrototype.

	Replications			Selection
*K*	Min.	Mean	Max.	LDAPrototype
*R* = 50				
5	0.8754	0.8988	0.9205	0.9657
6	0.8456	0.8696	0.9026	0.9626
7	0.8091	0.8394	0.8623	0.9542
8	0.7763	0.8043	0.8370	0.9340
9	0.7397	0.7740	0.8000	0.9157
10	0.7167	0.7473	0.7806	0.8967
11	0.7000	0.7220	0.7466	0.8784
12	0.6798	0.7029	0.7288	0.8707
13	0.6615	0.6844	0.7116	0.8596
14	0.6321	0.6625	0.6900	0.8369
15	0.6045	0.6415	0.6639	0.8066
*R* = 100				
5	0.8806	0.8975	0.9098	0.9834
6	0.8521	0.8689	0.8933	0.9688
7	0.8123	0.8376	0.8541	0.9681
8	0.7832	0.8035	0.8362	0.9490
9	0.7572	0.7752	0.7952	0.9313
10	0.7279	0.7479	0.7660	0.9189
11	0.7022	0.7224	0.7430	0.9129
12	0.6815	0.7019	0.7240	0.9018
13	0.6543	0.6832	0.7014	0.8788
14	0.6439	0.6647	0.6849	0.8626
15	0.6233	0.6436	0.6641	0.8407
*R* = 200				
5	0.8878	0.8975	0.9067	0.9861
6	0.8565	0.8684	0.8832	0.9780
7	0.8189	0.8372	0.8544	0.9771
8	0.7849	0.8065	0.8226	0.9613
9	0.7564	0.7752	0.7913	0.9351
10	0.7370	0.7473	0.7587	0.9242
11	0.7070	0.7229	0.7399	0.9303
12	0.6832	0.7017	0.7140	0.9204
13	0.6666	0.6828	0.6962	0.9005
14	0.6497	0.6645	0.6778	0.8768
15	0.6263	0.6431	0.6545	0.8498
*R* = 500				
5	0.8910	0.8985	0.9042	0.9912
6	0.8578	0.8699	0.8814	0.9857
7	0.8290	0.8375	0.8449	0.9799
8	0.7959	0.8053	0.8146	0.9679
9	0.7656	0.7745	0.7849	0.9492
10	0.7394	0.7483	0.7577	0.9334
11	0.7136	0.7240	0.7322	0.9360
12	0.6950	0.7025	0.7127	0.9538
13	0.6755	0.6825	0.6900	0.9209
14	0.6541	0.6635	0.6711	0.8942
15	0.6356	0.6429	0.6511	0.8739

## Additional Information and Declarations

### Data Availability

The following information was supplied regarding data availability: Software package ldaPrototype is available at Zenodo: Jonas Rieger. (2020). JonasRieger/ldaPrototype: ldaPrototype 0.3.0 (v0.3.0). Zenodo. https://doi.org/10.5281/zenodo.4302111
The data is available at Zenodo and Eldorado - Repository of the TU Dortmund: Jonas Rieger. (2020). JonasRieger/ldaPrototype: ldaPrototype 0.3.0 (v0.3.0). Zenodo. https://doi.org/10.5281/zenodo.4302111
Jonas Rieger, lkoppers, Kenneth Benoit, & karinboczek. (2020). Docma-TU/tosca: tosca 0.2-0 (v0.2-0). Zenodo. https://doi.org/10.5281/zenodo.3703066

http://dx.doi.org/10.17877/DE290R-21911. The computer code is available in the Supplemental Files.
